# A patient-level pooled analysis of treatment-shortening regimens for drug-susceptible pulmonary tuberculosis

**DOI:** 10.1038/s41591-018-0224-2

**Published:** 2018-11-05

**Authors:** Marjorie Z. Imperial, Payam Nahid, Patrick P. J. Phillips, Geraint R. Davies, Katherine Fielding, Debra Hanna, David Hermann, Robert S. Wallis, John L. Johnson, Christian Lienhardt, Rada M. Savic

**Affiliations:** 1 University of California, San Francisco, San Francisco, CA USA; 2 University of Liverpool, Liverpool, UK; 3 London School of Hygiene and Tropical Medicine, London, UK; 4 Critical Path Institute, Tucson, AZ USA; 5 Bill and Melinda Gates Foundation, Seattle, WA USA; 6 Aurum Institute and ACT4TB/HIV, Johannesburg, South Africa; 7 Case Western Reserve University, Cleveland, OH USA; 8 University Hospitals Cleveland Medical Center, Cleveland, OH USA; 9 Global Tuberculosis Programme, World Health Organization, Geneva, Switzerland; 10 Unité Mixte Internationale TransVIHMI (UMI 233 IRD–U1175 INSERM–Université de Montpellier), Institut de Recherche pour le Développement (IRD), Montpellier, France

**Keywords:** Therapeutics, Antimicrobial therapy, Tuberculosis, Public health, Risk factors

## Abstract

Tuberculosis kills more people than any other infectious disease. Three pivotal trials testing 4-month regimens failed to meet non-inferiority margins; however, approximately four-fifths of participants were cured. Through a pooled analysis of patient-level data with external validation, we identify populations eligible for 4-month treatment, define phenotypes that are hard to treat and evaluate the impact of adherence and dosing strategy on outcomes. In 3,405 participants included in analyses, baseline smear grade of 3+ relative to <2+, HIV seropositivity and adherence of ≤90% were significant risk factors for unfavorable outcome. Four-month regimens were non-inferior in participants with minimal disease defined by <2+ sputum smear grade or non-cavitary disease. A hard-to-treat phenotype, defined by high smear grades and cavitation, may require durations >6 months to cure all. Regimen duration can be selected in order to improve outcomes, providing a stratified medicine approach as an alternative to the ‘one-size-fits-all’ treatment currently used worldwide.

## Abstract (web summary)

Analysis of tuberculosis drug trials identifies features to stratify patients for longer or shorter treatment duration than the standard of care, in order to improve therapeutic outcomes.

## Main

Three recent international randomized phase 3 trials evaluating 4-month fluoroquinolone-containing regimens in adults with pulmonary, drug-susceptible tuberculosis failed to achieve non-inferiority compared with the standard 6-month control regimen (OFLOTUB^1^, ClinicalTrials.gov number NCT00216385; REMoxTB^2^, ClinicalTrials.gov number NCT00864383; RIFAQUIN^3^, ISRCTN number 44153044). These trials evaluated later-generation fluoroquinolones (gatifloxacin and moxifloxacin) as single substitutions for ethambutol or isoniazid in multidrug regimens with the objective of shortening treatment duration from 6 to 4 months. In each of the three trials, the 4-month regimen did not satisfy the criteria for non-inferiority. However, the experimental 4-month regimens did cure approximately four-fifths of the participants, suggesting that a large proportion of global tuberculosis cases could be successfully treated with shorter duration^1–3^.

Since the introduction of highly effective rifampin-based regimens in the 1970s and 1980s, the treatment of tuberculosis has been a ‘one-size-fits-all’ paradigm, with a 6-month regimen composed of four drugs (isoniazid, rifampin, pyrazinamide and ethambutol) used for all patients with drug-susceptible pulmonary tuberculosis^4,5^. Regimen administration is coupled with various adherence interventions at the programmatic level, including directly observed therapy, to ensure regimen intake^4^. In programs, the one-size-fits-all paradigm leads to undertreatment of patients with severe forms of disease and entails unnecessarily long treatment with potential toxicities for many patients in whom there is a lower disease burden, which in turn may result in increased rates of loss to follow-up^6^. In clinical trials, one-size-fits-all experimental regimens have been consistently inadequate to cure the hardest-to-treat tuberculosis patients, indicating that treatment duration is a critical determinant for cure^7^. Moreover, even for the standard 6-month regimen, the recent trials demonstrate that 5–8% of patients fail treatment or relapse and 15–20% experience composite unfavorable outcomes^1–3,8^. Tuberculosis is not a uniform clinical entity; it presents with wide variation in severity of disease at the time of diagnosis. Yet current tuberculosis regimen development efforts are aimed at using new drugs with increased potency to identify shorter treatments for all patients, regardless of severity of disease. This approach places otherwise efficacious drugs and regimens at risk of being abandoned, consequently impeding the identification of new tuberculosis regimens that would be curative if used with greater precision.

In this pooled analysis of individual participant datasets from these high-quality, contemporary trials, we sought to identify characteristics of those participants who were cured with 4-month regimens and, conversely, of those with hard-to-treat phenotypes of tuberculosis, who might require longer treatment durations. We evaluated both baseline characteristics and on-treatment markers of risk, including dosing frequency and adherence, for their ability to stratify the study population into easy- or hard-to-treat phenotypes of tuberculosis.

## Results

### Study participants

**Fig. 1:**
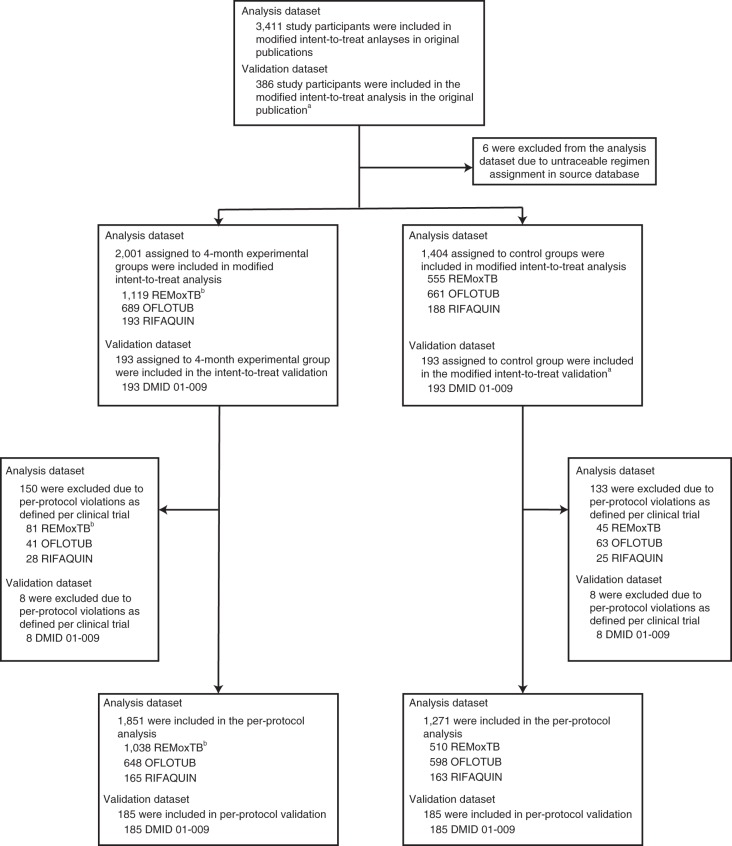
Analysis and validation populations. Individual participant data from three trials were pooled for analysis. The original results were published in ref. ^1^ (OFLOTUB), ref. ^2^ (REMoxTB) and ref. ^3^ (RIFAQUIN). Data from a fourth trial, DMID 01-009, were used for external validation and previously published in ref. ^9^. The modified intent-to-treat population was used for the analysis. ^a^For the validation dataset, the time-to-event analysis population in the original publication was used. ^b^REMoxTB included two 4-month experimental groups.

A total of 3,411 study participants treated for drug-susceptible tuberculosis with one of four fluoroquinolone-containing 4-month regimens (*n* = 2,001) or the standard 6-month regimen (*n* = 1,404) were included in the modified intent-to-treat analyses of the OFLOTUB^1^, REMoxTB^2^, and RIFAQUIN^3^ trials; 6 participants were excluded from the current analyses due to inability to verify treatment allocation in source databases. The external validation dataset (DMID 01-009; see ref. ^9^) includes 193 study participants treated with a 4-month experimental regimen (no fluoroquinolone) and 193 study participants treated with the standard 6-month regimen (Fig. 1). Baseline characteristics of participants did not differ across the experimental and control groups within analysis datasets, with the exception of race and enrollment at sites in the country of Senegal (both *P* < 0.001; Table 1); 12% of the participants were infected with HIV.

**Table 1:** Baseline characteristics of study participants in the modified intent-to-treat analysis

	Analysis dataset (OFLOTUB, REMoxTB, RIFAQUIN)		Validation dataset (DMID 01-009)	
	Experimental group (*n* = 2,001)	Control group (*n* = 1,404)	Experimental group (*n* = 193)	Control group (*n* = 193)
**Country (no. of participants (%))**				
Benin	122 (6)	108 (8)	–	–
Botswana	11 (<1)	12 (<1)	–	–
China	12 (<1)	8 (<1)	–	–
Guinea	191 (10)	184 (13)	–	–
India	228 (11)	114 (8)	–	–
Kenya	165 (8)	122 (9)	–	–
Malaysia	43 (2)	20 (1)	–	–
Senegal	129 (6)	138 (10)	–	–
South Africa	811 (41)	516 (37)	–	–
Tanzania	122 (6)	67 (5)	–	–
Thailand	65 (3)	34 (2)	–	–
Zambia	35 (2)	21 (1)	–	–
Zimbabwe	67 (3)	60 (4)	–	–
Brazil	–	–	67 (35)	68 (35)
Philippines	–	–	46 (24)	46 (24)
Uganda	–	–	80 (41)	79 (41)
S**ex (no. of participants (%))**				
Female	592 (30)	415 (30)	76 (39)	76 (39)
**Race (no. of participants (%))** ^**a**^				
Black or African American	1,326 (66)	1,066 (76)	–	–
Asian	349 (17)	178 (13)	–	–
Other	326 (16)	160 (11)	–	–
**Age (years)** ^**b**^				
Median	30	29	29	27
Interquartile range	24–39	24–38	23–38	22–36
**Weight (kg)**				
Median	52	52	54	55
Interquartile range	46–58	47–58	49–62	49–61
**BMI (kg** **m**^**−2**^**)**^**c**^				
Median	18.4	18.3	20.3	19.5
Interquartile range	16.9–20.2	16.9–20.1	18.7–22.2	18.5–22.0
**HIV status (no. of participants (%))** ^**d**^				
HIV positive	248 (12)	220 (16)	0 (0)	0 (0)
**CD4** ^**+**^ **cell count** ^**e**^				
Median	363	317	–	–
Interquartile range	265–493	241–444	–	–
≤300 (no. of participants)	74	81	–	–
>300 (no. of participants)	135	99	–	–
**Cavitation (no. of participants (%))** ^**f**^				
Cavitation present	1,247 (62)	847 (60)	0 (0)	0 (0)
**Smear (no. of participants (%))** ^**g**^				
Negative	151 (8)	85 (6)	85 (44)	85 (44)
1+	332 (17)	232 (17)	26 (14)	30 (15)
2+	503 (25)	404 (29)	32 (17)	36 (18)
3+	988 (49)	667 (48)	50 (26)	42 (22)

^a^Race was missing for all OFLOTUB study participants; black race was assigned to all study participants given all OFLOTUB sites were in Africa.

^b^Age was missing for 5 study participants.

^c^BMI was defined as the weight in kilograms divided by the squared height in meters. Height was missing for 291 study participants; median height for females and males were used to calculate BMI for those participants.

^d^HIV status was missing for 9 study participants.

^e^CD4^+^ cell count cutoff was variable across trials (described in Supplementary Table 2). CD4^+^ cell count summary statistics were based only on study participants co-infected with HIV but were missing for 79 HIV co-infected study participants.

^f^Cavitation status was missing for 200 study participants.

^g^Smear grade was based on clinical trial-defined grading but readjusted so all data were on the same scale. Smear grade was missing for 43 study participants.

### Primary outcome analysis

**Fig. 2:**
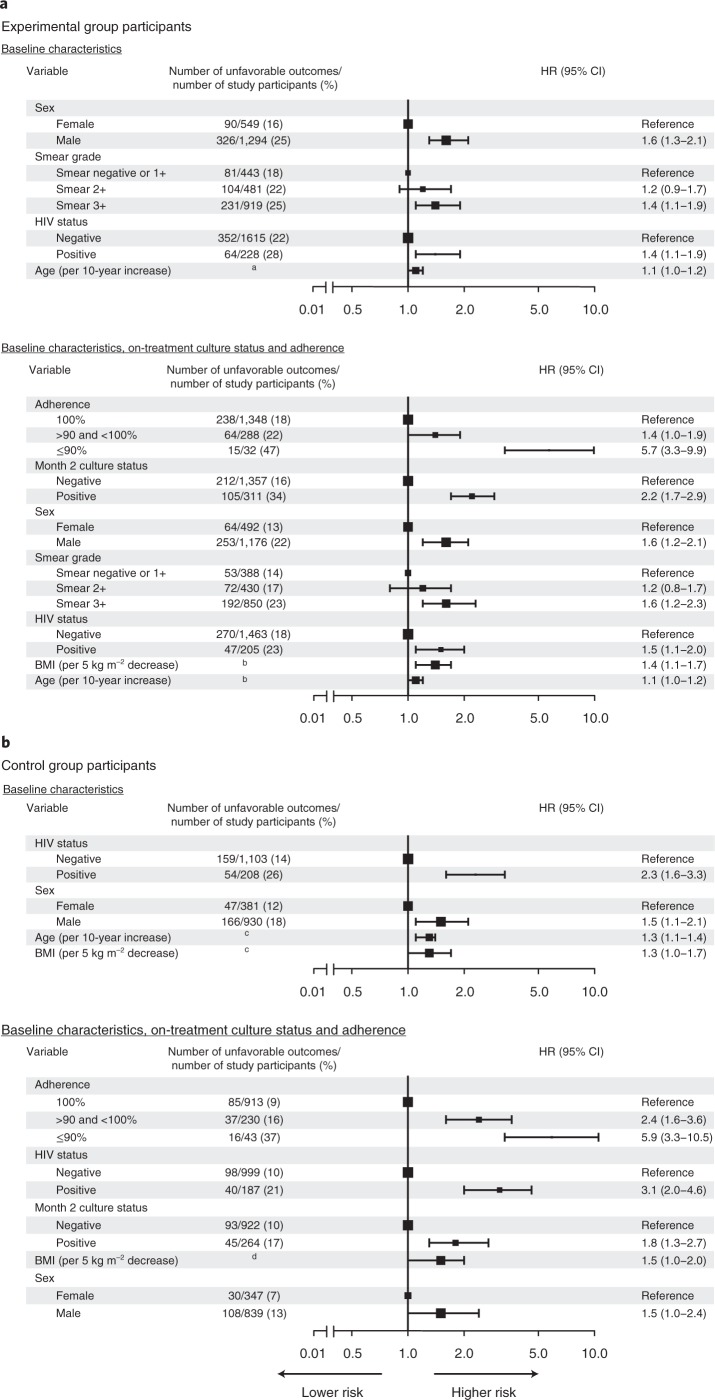
Multivariate HRs for unfavorable outcomes. **a**, Multivariate analysis for experimental group with baseline predictors (top) and baseline and on-treatment predictors (bottom). **b**, Multivariate analysis for control group with baseline predictors (top) and baseline and on-treatment predictors (bottom). All analyses were adjusted for country, and effect sizes are available in Supplementary Tables 4 and 5. HRs with 95% Wald CIs are reported. The size of the square denotes the relative sample size according to variable. ^a^Age <30 years, 179/916 (20%) unfavorable outcomes and age ≥30 years, 237/927 (26%) unfavorable outcomes. ^b^Age <30 years, 136/830 (16%) unfavorable outcomes and age ≥30 years, 181/838 (22%) unfavorable outcomes; BMI ≥17 kg m^−2^, 226/1,247 (18%) unfavorable outcomes and BMI <17 kg m^−2^, 91/421 (22%) unfavorable outcomes. ^c^Age <30 years, 92/657 (14%) unfavorable outcomes and age ≥30 years, 121/654 (19%) unfavorable outcomes; BMI ≥17 kg m^−2^, 156/989 (16%) unfavorable outcomes and BMI <17 kg m^−2^, 57/322 (18%) unfavorable outcomes. ^d^BMI ≥17 kg m^−2^, 102/901 (11%) unfavorable outcomes and BMI <17 kg m^−2^, 36/285 (13%) unfavorable outcomes.

Multivariate Cox analysis of baseline risk factors for unfavorable outcomes included 3,154 of 3,405 participants (93%) with no missing baseline covariates; 1,843 of 2,001 participants (92%) were allocated to one of the 4-month experimental regimens, and 1,311 of 1,404 participants (93%) were allocated to the control regimens (Supplementary Tables 1–3). In participants assigned to 4-month experimental regimens, baseline smear grade of 3+ relative to negative or 1+ grade and HIV seropositivity were the two major baseline clinical risk factors for unfavorable outcomes, with an adjusted hazard ratio (HR) of 1.4 (95% confidence interval (CI), 1.1–1.9) and 1.4 (95% CI, 1.1–1.9), respectively, adjusted also for age and sex. Higher risk was observed in older participants (adjusted HR, 1.1 per 10 years increase; 95% CI, 1.0–1.2) and male participants (HR, 1.6; 95% CI, 1.3–2.1). After inclusion of on-treatment culture and adherence as risk factors, 1,668 of 2,001 experimental arm participants (83%) were available for analysis. Non-adherence was the most significant risk factor for unfavorable outcome, with adjusted HRs of 5.7 (95% CI, 3.3–9.9) for participants who missed 10% or more prescribed doses and 1.4 (95% CI, 1.0–1.9) for participants who missed less than 10% of prescribed doses relative to participants who completed treatment without any missed doses. Month 2 culture positivity was significantly associated with unfavorable outcome (HR, 2.2; 95% CI, 1.7–2.9). After adjustment for on-treatment factors, lower body mass index (BMI, representative of malnutrition) was a risk factor for unfavorable outcome (HR, 1.4 per 5 kg m^−2^ decrease; 95% CI, 1.1–1.7) (Fig. 2a and Supplementary Table 4).

In the 1,311 of 1,404 participants (93%) allocated to the 6-month control regimen, HIV seropositivity was the most significant baseline risk factor for unfavorable outcomes, with an adjusted HR of 2.3 (95% CI, 1.6–3.3). Participants who were older (HR, 1.3 per 10 years increase; 95% CI, 1.1–1.4), were male (HR, 1.5; 95% CI, 1.1–2.1) or had lower BMI at study entry (HR, 1.3 per 5 kg m^−2^ decrease; 95% CI, 1.0–1.7) had higher risk of unfavorable outcomes. Of control-arm participants, 1,186 of 1404 (84%) contributed data both for baseline and on-treatment risk factors. Non-adherence was the most significant on-treatment risk factor for unfavorable outcomes, with adjusted HR of 5.9 (95% CI, 3.3–10.5) for participants who missed 10% or more and 2.4 (95% CI, 1.6–3.6) for participants who missed less than 10% of prescribed doses relative to participants who completed treatment without any missed doses. On-treatment culture positivity was also identified as a significant risk factor for unfavorable outcomes (month 2 HR, 1.8; 95% CI, 1.3–2.7). After adjustment for on-treatment factors, HIV positivity (HR, 3.1; 95% CI, 2.0–4.6), male sex (HR, 1.5; 95% CI, 1.0–2.4), and lower BMI (HR, 1.5 per 5 kg m^−2^ decrease; 95% CI, 1.0–2.0) remained as factors associated with high risk (Fig. 2b and Supplementary Table 5). In the per-protocol analysis, results were similar in the experimental and control groups when compared with the primary modified intent-to-treat analysis (Supplementary Table 6).

### Non-inferiority test

**Fig. 3:**
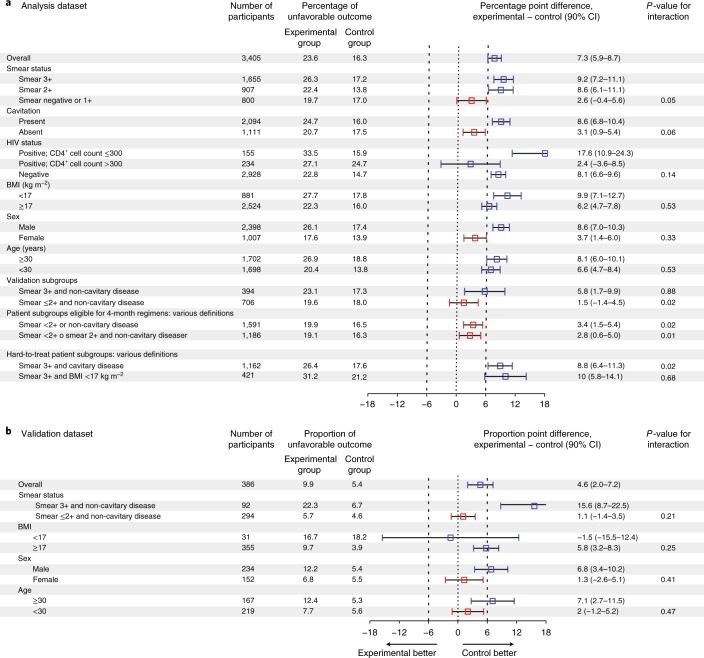
Difference in percentage of unfavorable outcomes between the experimental group and the control group, overall and according to subgroups. **a**, Non-inferiority tests based on analysis dataset. **b**, Validation of non-inferiority tests in **a** based on an independent validation dataset. The 90% CIs of the differences in percentage of unfavorable outcomes were determined by bootstrapping 500 samples. Red squares denote experimental subgroups that were non-inferior to the control subgroups, and blue squares denote subgroups that did not show non-inferiority. Study participants in the validation dataset were HIV-uninfected adults with non-cavitary disease and month 2 culture-negative status.

The percentage of unfavorable outcomes at 24 months for study participants with a baseline negative or 1+ grade smear was similar in experimental and control regimens, indicating non-inferiority (difference in study adjusted Kaplan–Meier estimate of unfavorable outcome, 2.6; 90% CI, −0.4 to 5.6; *P* = 0.05 for interaction). Additionally, study participants with non-cavitary disease had a similar percentage of unfavorable outcomes between experimental and control regimens (difference in study adjusted Kaplan–Meier estimate of unfavorable outcome, 3.1; 90% CI, 0.9–5.4; *P* = 0.06 for interaction). In an easy-to-treat phenotype of tuberculosis consisting of participants with 1+ or negative smear or non-cavitary disease that constituted 47% of the study population (1,591 of 3,405 participants), the 4-month regimens were non-inferior to the 6-month control regimen (Fig. 3a). In a hard-to-treat phenotype of tuberculosis consisting of participants with 3+ smear and cavitary disease that constituted 34% of the study population (1,162 of 3,405 participants), the 4-month regimens were clearly inferior.

### External validation

Using an independent dataset available from the DMID 01-009 trial in patients with non-cavitary disease, the patient population eligible for a 4-month rifampin-containing regimen was validated, confirming that for study participants with low to moderate smear grade, a standard regimen shortened to 4 months was non-inferior to the standard 6-month regimen. We confirmed that study participants with high smear grade were the driver of high rates of unfavorable outcomes in the 4-month DMID 01-009 regimen (Fig. 3b).

### Impact of dosing frequency

**Fig. 4:**
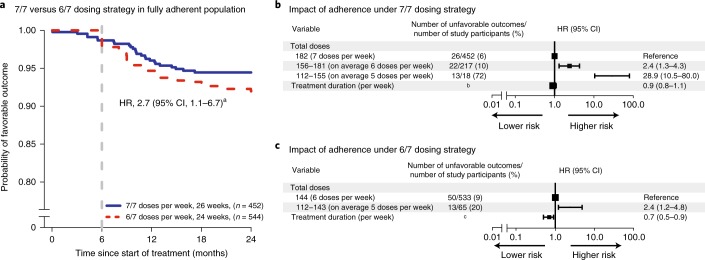
Analysis of 7/7 and 6/7 dosing strategies and impact of adherence in the control group. **a**, Kaplan–Meier estimates for fully adherent study participants (*n* = 996) after treatment with 7/7 or 6/7 dosing strategies. **b**, Multivariate analysis with total number of doses taken for study participants who took at least 4 months of treatment under 7/7 dosing strategies for 26 weeks (REMoxTB and RIFAQUIN trials), after adjustment for country and treatment duration. **c**, Multivariate analysis with total number of doses taken for study participants who took at least 4 months of treatment under 6/7 dosing strategies for 24 weeks (OFLOTUB trial), after adjustment for country and treatment duration. Effect sizes for country are available in Supplementary Table 7. In **b** and **c**, the HRs with 95% Wald CI are reported. ^a^Hazard ratio with 95% Wald CI for 6/7 dosing strategy relative to 7/7 dosing strategy for fully adherent population after adjustment for country and treatment duration. ^b^Treatment duration <182 days, 21/110 (19%) unfavorable outcomes and treatment duration ≥182 days, 40/577 (7%) unfavorable outcomes. ^c^Treatment duration <169 days, 21/155 (14%) unfavorable outcomes and treatment duration ≥169 days, 42/443 (9%) unfavorable outcomes.

Kaplan–Meier estimates show that study participants who fully adhered to a dosing regimen of 6 of 7 days per week (6/7) had a higher probability of unfavorable outcome than those who adhered to and completed a dosing regimen of 7 of 7 days per week (7/7) (HR, 2.7; 95% CI, 1.1–6.7, after adjustment for treatment duration and country) (Fig. 4a).

To assess the impact of partial adherence on standard of care under a 7/7 or 6/7 dosing strategy, 1,285 participants who completed at minimum 4 months of treatment (112 total doses) were included in the Cox regression analysis. This analysis set included 687 participants who were prescribed treatment with a 7/7 weekly dosing strategy for 26 weeks (REMoxTB and RIFAQUIN trials) and 598 participants who were prescribed treatment with a 6/7 weekly dosing strategy for 24 weeks (OFLOTUB trial). On a 7/7 weekly dosing strategy for 26 weeks, participants who took 156 to 181 total doses (corresponding to an average of 6 doses per week, or missing up to 14% pills) or 112 to 155 total doses (corresponding to an average of 5 doses per week, or missing 14–33% pills) had significantly higher risk of unfavorable outcomes relative to those who took all 182 prescribed doses (7 doses per week), with HRs of 2.4 (95% CI, 1.3–4.3) and 28.9 (95% CI, 10.5–80.0), respectively, adjusted for treatment duration and country (Fig. 4b and Supplementary Table 7). Similarly, participants receiving 112 to 143 doses (average of 5 doses per week) had a higher risk of unfavorable outcomes relative to those who took the complete 144 prescribed doses (6 doses per week) for 24 weeks, with HR of 2.4 (95% CI, 1.2–4.8), adjusted for treatment duration and country (Fig. 4c and Supplementary Table 7).

## Discussion

In this pooled analysis of individual participant data from recent phase 3 treatment-shortening trials, we have shown that adult patients with minimal disease, as defined by low smear grade or the absence of cavitation, were at lower baseline risk for unfavorable outcomes; in this population the experimental 4-month regimens are effective. Patients with either of these low-risk characteristics, which define an easy-to-treat phenotype of tuberculosis, constituted 47% of the total study population (1,591 of 3,405 participants). Conversely, we have shown that a smear grade of 3+ and the presence of cavitation on chest radiographs at baseline define a hard-to-treat phenotype, constituting 34% of the study population (1,162 of 3,405 participants), and this group may require longer durations of treatment than the current standard 6-month regimen to achieve the highest cure rates feasible. In our analyses, other baseline characteristics associated with unfavorable outcomes included being HIV infected and having a lower BMI at study entry. Male sex was consistently and independently linked with poor likelihood of cure in both control and experimental regimens. The etiology for this association is not clear, particularly given that the association persists after adjusting for severity of disease and adherence. Our definitions of tuberculosis phenotypes were validated in an independent trial dataset of patients with non-cavitary disease. Whereas this trial was stopped early due to higher rates of unfavorable outcomes in the experimental 4-month regimen, we confirmed that a 4-month regimen would be effective for patients with negative, 1+ or 2+ smears in non-cavitary disease at baseline. We also confirmed that participants with high smear grade (3+) at baseline were more likely to fail treatment regardless of whether they received 4- or 6-month regimens, compared with those with lower smear grades at baseline. Given the established importance of cavitation in disease prognosis and treatment response^5,10,11^, we included this characteristic in the analyses of non-inferiority for various subgroups, despite the fact that cavitation was not a significant variable in the multivariate analysis and was only marginally significant in the univariate analysis (Supplementary Tables 8–10). In analyses limited to the trials providing detailed chest radiograph readout data, specifically OFLOTUB and RIFAQUIN, we confirmed that cavity size, bilateral disease and disease extent measured by zone scores were all significant risk factors for unfavorable outcome (Supplementary Figs. 1 and 2), confirming that disease severity determined by chest radiograph remains an important tool for the definition of hard-to-treat phenotypes and prediction of treatment outcome. Overall, we showed that the combination of smear grading and cavitary status adequately defines easy-to-treat and hard-to-treat groups; however, we also identified subgroups that allow for stratification when chest radiographic information is not available.

In this study, we also found that across both experimental and standard control regimens, minimal non-adherence and missed doses were associated with significantly increased risk for unfavorable outcome. Missing as few as one in ten doses of a regimen was associated with a fivefold increase in risk. Missed doses had a stronger association with poor outcome than did failure to achieve culture conversion at 2 months. Consistent with our analyses of non-adherence, dosing frequencies of less than 7/7 increase the chances of unfavorable outcome, even if participants are fully adherent (Fig. 4a). Current US tuberculosis treatment guidelines state, on the basis of clinical experience and program practicality, that drug administration 5 days per week is an acceptable alternative to administration 7 days per week, and that either approach may be considered as meeting the definition of ‘daily’ dosing^5^. Our findings suggest otherwise and provide data-driven evidence to support the use of 7/7 dosing^12,13^. The finding that the current rifampin-based regimen used worldwide has ‘low forgiveness’ for non-adherence or missed doses has important implications for tuberculosis care as well as for future design and conduct of clinical trials. A regimen with excellent efficacy under rigorous clinical trial settings that is otherwise unforgiving of missed doses will fail in the field. New and improved adherence interventions for tuberculosis have been introduced to facilitate treatment completion^14,15^; however, such tools can be limited by issues of scale-up, generalizability and cost. A more durable and patient-centered solution is the targeted development of regimens composed of drugs with long half-lives and steady pharmacokinetic profiles that will accommodate less than perfect adherence patterns in the field without penalty to the efficacy of the regimen. Our findings in this regard highlight the critical value of conducting pragmatic clinical trials that assess the effectiveness and robustness of regimens under programmatic conditions.

In this study, we found that the 4-month fluoroquinolone-containing regimens met the margin for non-inferiority in participants with a negative or 1+ grade baseline smear or non-cavitary disease. Conversely, we found that a hard-to-treat phenotype of tuberculosis defined by high smear grades and cavitation on baseline chest radiograph was associated with unfavorable outcomes. Randomized trials conducted by the British Medical Research Council largely in the pre-HIV era have previously illustrated that the majority of patients do not need 6 months of standard therapy^16,17^. Our analyses support this position and suggest that the current one-size-fits-all model of care leads to undertreatment of patients with severe forms of disease and to unnecessarily long treatment (with unjustified risk of drug toxicity) for many patients with less extensive disease. We believe our results provide justification to evaluate a stratified approach to tuberculosis therapeutics. By using baseline markers to determine the optimal stratum for a given patient, with decisions for treatment extension further enhanced by the use of on-treatment measures of adherence and clinical, microbiologic and radiographic markers, the feasibility of achieving cure in all patients with tuberculosis, rather than a majority, is enhanced. Pursuit of the highest possible cure rates in tuberculosis is an important public health priority, and perhaps more important than treatment shortening, as suggested by recent modeling work that shows increases in treatment efficacy will have the greatest impact on reducing mortality and burden of disease worldwide^18^. The tools necessary for using stratified medicine approaches to tuberculosis care at the program level are already in use in many settings, including HIV testing, CD4^+^ cell counts for HIV-positive patients, chest radiography, smear microscopy, and scales for measuring height and weight for calculation of BMI. Future trials that test stratified medicine approaches to tuberculosis care should also evaluate newer tools (for example, GeneXpert cycle threshold), which in turn would allow for algorithms for selecting duration to be further refined, offering additional characteristics and options for determining risk. Nonetheless, some patients will have limited access to these diagnostics, and in such settings, either a simpler stratification algorithm can be developed (for example, smear grade and BMI, as shown in Fig. 3a) or the currently used one-size-fits-all approach may still remain the most practical and implementable option.

Our study has limitations. Data sharing principles are supported in the tuberculosis therapeutics field^19,20^; however, data collection was not standardized across the included trials. Future protocols should use minimum dataset standards, compliant with Clinical Data Interchange Standards Consortium standards (https://www.cdisc.org), to allow robust pooled analyses. Second, chest radiograph interpretation was not uniform, and as such, we could not analyze size and number of cavities in all three studies (Supplementary Figs. 1 and 2). Third, very few pharmacokinetic data were available, hampering our ability to explore dosing, drug exposure and outcome relationships. We advocate for the inclusion of population pharmacokinetics in phase 3 trials to address the variability in responses across geographic regions and populations. Our comparison of 6/7 with 7/7 dosing was a comparison between trials rather than within trials and therefore may be confounded by other study differences. Finally, only 12% of participants had HIV co-infection, and many were not on effective antiretroviral therapy regimens; thus, caution should be used in generalizing our findings to immunocompromised populations. Strengths of our analyses include the inclusion of large datasets from four international registration-quality phase 3 trials conducted across diverse human populations in high-tuberculosis-burden settings in South America, sub-Saharan Africa and Asia; performance of microbiologic assays by quality-controlled laboratories; and the careful recording of study treatment under direct observation.

In sum, our validated analyses of individual participant data from contemporary randomized clinical trials provide three major findings. First, we show that low smear grades at baseline or the absence of cavitation identifies a population at low risk for recurrence in whom 4-month rifampin-containing regimens may be effective. Conversely, high sputum smear grade at baseline in conjunction with the presence of cavities defines a hard-to-treat phenotype that may require longer durations of treatment than the current standard of care to achieve high cure rates. There is also a third phenotype, made up of the remaining patients for whom treatment shortening may also be possible. Second, we show that minor degrees of non-adherence or missed doses significantly increase the risk for poor outcomes. Third, we show that simple baseline and on-treatment markers could be used to select treatment duration with greater precision, providing a programmatically viable alternative to the one-size-fits-all paradigm used worldwide. Our results indicate that stratified medicine principles should be further evaluated in clinical trials of tuberculosis therapeutics.

## Methods

### Study design

This study utilized individual participant data from four recent, international, randomized phase 3 trials (OFLOTUB, REMoxTB, RIFAQUIN and DMID 01-009)^1–3,9^ that compared 4-month regimens to standard 6-month regimens endorsed by the World Health Organization and the American Thoracic Society/Centers for Disease Control and Prevention/Infectious Diseases Society of America for drug-susceptible pulmonary tuberculosis^4,5^. The OFLOTUB trial compared an experimental 4-month gatifloxacin-based regimen with a 6-month standard regimen^1^. The REMoxTB trial compared two experimental 4-month moxifloxacin-based regimens to a 6-month standard regimen^2^. The RIFAQUIN trial compared experimental 4- or 6-month moxifloxacin- and high-dose-rifapentine-based intermittent regimens with a 6-month standard regimen^3^. A fourth independent tuberculosis treatment-shortening trial sponsored by the National Institute of Allergy and Infectious Diseases and conducted by the National Institutes of Health-funded Tuberculosis Research Unit compared a 4-month standard regimen (with no fluoroquinolone) with a 6-month standard regimen in adults with non-cavitary disease and 2-month negative culture status (DMID 01-009; ClinicalTrials.gov number NCT00130247)^9^. The pooled analyses are focused on data from participants receiving the 4-month experimental regimens and 6-month standard regimens and do not include the once-weekly (in continuation phase) fluoroquinolone 6-month experimental regimen in the RIFAQUIN trial. The three trials that compared four fluoroquinolone-based tuberculosis regimens to a 6-month standard regimen provided data for identifying markers and models for risk stratification, while the DMID 01-009 trial data were used for external validation. We defined the experimental group as all study participants allocated to any of the 4-month experimental regimens and the control group as all study participants allocated to the 6-month standard regimen. The protocol for each study was reviewed and approved by ethics committees and regulatory committees described in the original publications, and all participants provided written informed consent^1–3,9^.

### Data acquisition, management and harmonization

Integrated and standardized individual-level data in each of the trials were obtained through the Platform for Aggregation of Clinical TB Studies (TB-PACTS; https://c-path.org/programs/tb-pacts/). Data sharing was directed by comprehensive data contribution agreements with sponsors. Before data were pooled, we compared trial protocols, case report forms and data dictionaries to harmonize databases. Data queries were resolved through direct consultations with each trial team and Critical Path Institute data managers. After pooling data, data inputs were checked for missing or duplicated values, for consistency and for plausibility. Final dataset specification is available in Supplementary Tables 1 and 2, and access to original databases is available through TB-PACTS. Data from DMID 01-009 were obtained directly from the sponsor. Further information on data acquisition and availability is described in the Life Sciences Reporting Summary.

### Efficacy outcomes

The primary efficacy endpoint of the pooled analysis was time to an unfavorable outcome for a maximum of 24 months after start of treatment (OFLOTUB study participants were followed until 24 months after start of treatment, and RIFAQUIN and REMoxTB study participants were followed for 18 months), as defined according to each trial protocol and described in the original publications. Trial-specific definitions of unfavorable outcome were broadly similar but included some differences, which are outlined in Supplementary Table 1. For example, re-infections confirmed by mycobacterial interspersed repetitive unit (MIRU) typing were excluded from the composite definition of unfavorable outcome in the primary analysis of the REMoxTB and RIFAQUIN trials, whereas they were included in the composite definition of unfavorable outcome in the primary analysis of the OFLOTUB trial. Sensitivity analyses were performed to evaluate the inclusion of all MIRU-confirmed re-infections, classified as unfavorable (Supplementary Table 11) or favorable or completely removed from the analysis. The secondary efficacy outcome was the non-parametric Kaplan–Meier estimate of unfavorable outcome at 24 months after start of treatment.

### Baseline predictors

The primary analysis set included baseline predictors, which were missing in no more than 10% of participants: age, race, BMI, sex, presence of cavitation on chest radiograph and smear grade (Supplementary Table 2). Weight was also considered for inclusion in the primary analysis but ultimately was not included due to its moderate correlation with BMI (Spearman coefficient, 0.74; Supplementary Fig. 3). No major covariate imputation was done, with two exceptions: (1) black race was assigned for all participants in the OFLOTUB trial, in which race information was not available, given that all OFLOTUB sites were in Africa and similar demographic characteristics were observed in other studies at their African sites (majority black); (2) median height for females and males of available data was used for 291 participants with missing height to calculate BMI, defined as the weight in kilograms divided by the squared height in meters (additional details available in Supplementary Table 2). Smear grading was specific for each microscopy method, each study and, in the RIFAQUIN trial, each study center (described in study protocols and lab manuals)^1–3,9^. The RIFAQUIN and OFLOTUB trials reported smear grade using a negative, 1+, 2+ and 3+ system, while the REMoxTB and the validation study reported smear grade using a 1+, 2+, 3+ and 4+ system. A conversion chart available in the REMoxTB trial lab manual was used to synchronize all smear data to the same grading scale^2^. Additional participant characteristics (smoking, cough grade and other radiographic measures) were considered but not included in the primary analysis due to large proportions of missing data (>10%; Supplementary Table 2).

### On-treatment predictors

On-treatment culture time point universally applied in all trials was month 2 culture status on Lowenstein–Jensen (LJ) solid medium or in liquid medium using the mycobacteria growth indicator tube (MGIT) system. Culture positivity on either medium was used for analyses, with preference for solid culture if available. Univariate Cox proportional hazard analysis for merged MGIT and LJ culture data (as described above), MGIT data only and LJ data only showed similar results in each treatment group (Supplementary Table 12). Treatment adherence was calculated as the number of days that doses were taken divided by the prescribed number of days. For participants with an unfavorable event during the treatment phase, the adherence calculation was adjusted for duration completed; for example, full adherence was assigned for study participants who took all doses up to the time of the event if the event appeared during treatment.

Individuals with missing data between the predefined sets of predictors were excluded from the multivariate analysis (summary on analysis populations available in Supplementary Table 3). There were no major correlations between the predefined sets of baseline and on-treatment predictors (Supplementary Fig. 3).

### Statistical analysis

All analyses were conducted using modified intent-to-treat and per-protocol populations, with the former used for primary analysis (per-protocol results summarized in Supplementary Table 6). Definitions for analysis populations are provided in the clinical trial protocols^1–3,9^.

To identify risk factors of time to unfavorable outcomes, we performed multivariate Cox proportional hazards analysis. HRs with 95% Wald CIs were reported. Analyses were conducted separately for the experimental and control regimens, as the hard-to-treat phenotypes may be different for different treatment durations. All multivariate analyses were adjusted for study country. The proportional hazard assumption was tested using Schoenfeld residuals, with a *P* < 0.05 for non-proportionality. Model selection for multivariate Cox analysis started with a full model (included all predefined predictors) that was followed by a backward stepwise approach (*P* > 0.05 to remove), then a forward stepwise approach to test predictors that were removed in the backward step (*P* < 0.01 to include). Predictors were included using linear relationships. Non-inferiority analyses were performed in study participant subgroups, according to identified risk factors in the multivariate Cox analysis. The test for interaction for each subgroup was performed prior to non-inferiority subgroup tests^21^. The absolute difference in percentage of unfavorable outcomes was calculated using inverse probability study weighted Kaplan–Meier estimates^22^ at 24 months after start of treatment to include maximal patient-years of follow-up and retain maximal data. Non-inferiority was assessed using the upper bound of the two-sided 90% CI, determined by bootstrapping 500 samples, and a non-inferiority margin of 6 percentage points, which was used in all the parent trials^1–3^.

Further analyses were performed to assess the impact of 7/7 (REMoxTB and RIFAQUIN) and 6/7 (OFLOTUB) weekly dosing strategies on outcomes. First, we compared Kaplan–Meier estimates for 7/7 and 6/7 weekly dosing strategies in study participants who completed their prescribed treatment. Second, we performed separate Cox proportional hazards analyses for trials with different weekly dosing strategies and assessed total number of days that the drugs were taken (total doses) and treatment duration (time between first and last dose dates) as predictors of treatment outcomes. To allow for pragmatic interpretation, HRs were reported for total doses of 156 to 181 (on average 6/7 doses per week) and 112 to 155 (on average 5/7 doses per week) relative to 182 (on average 7/7 doses per week) for the REMoxTB and RIFAQUIN analysis (7/7 weekly dosing strategies for 26 weeks). For the OFLOTUB analysis (6/7 weekly dosing strategy for 24 weeks), HRs were reported for total doses of 112 to 143 (on average 5/7 doses per week) relative to 144 (on average 6/7 doses per week). We have used an arbitrarily lower cutoff of 112 total doses, as it coincides with 4 months of treatment on a 7/7 dosing strategy and most of the data were clustered above this cutoff point. We have performed sensitivity analysis with cutoffs of at least 130 (exact number of doses if participant took 5/7 doses for 26 weeks) for the REMoxTB and RIFAQUIN analysis and 120 (exact number of doses if participant took 5/7 doses for 24 weeks) for the OFLOTUB analysis. Each analysis was adjusted for study country.

All data management, analyses and visualization were performed using R statistical software (version 3.4.3; https://www.r-project.org/).

### Reporting Summary

Further information on research design is available in the Nature Research Reporting Summary linked to this article.

## Online content

Any methods, additional references, Nature Research reporting summaries, source data, statements of data availability and associated accession codes are available at 10.1038/s41591-018-0224-2

## Supplementary Information

Supplementary Text and Figures Supplementary Figures 1–3 and Supplementary Tables 1–12

Reporting Summary

## Data Availability

The standardized data for the OFLOTUB (ClinicalTrials.gov number NCT00216385), REMoxTB (ClinicalTrials.gov number NCT00864383), and RIFAQUIN (ISRCTN number 44153044) trials that support the findings of this study are publicly available to qualified researchers through the Platform for Aggregation of Clinical TB Studies (TB-PACTS; https://c-path.org/programs/tb-pacts/). The DMID 01-009 (ClinicalTrials.gov number NCT00130247) data that support the findings of this study are available from the Tuberculosis Research Unit at Case Western Reserve University, but restrictions apply to the availability of these data, which were used under agreement for the current study. Data are however available from the authors upon reasonable request and with permission from the Tuberculosis Research Unit.

## References

[1] Merle CS (2014). A four-month gatifloxacin-containing regimen for treating tuberculosis. N. Engl. J. Med..

[2] Gillespie SH (2014). Four-month moxifloxacin-based regimens for drug-sensitive tuberculosis. N. Engl. J. Med..

[3] Jindani A (2014). High-dose rifapentine with moxifloxacin for pulmonary tuberculosis. N. Engl. J. Med..

[4] World Health Organization. *Guidelines for Treatment of Drug-Susceptible Tuberculosis and Patient Care, 2017 Update* (World Health Organization, Geneva, 2017).

[5] Nahid P (2016). Official American Thoracic Society/Centers for Disease Control and Prevention/Infectious Diseases Society of America clinical practice guidelines: treatment of drug-susceptible tuberculosis. Clin. Infect. Dis..

[6] Jo K-W (2014). Risk factors for 1-year relapse of pulmonary tuberculosis treated with a 6-month daily regimen. Respir. Med..

[7] Benator D (2002). Rifapentine and isoniazid once a week versus rifampicin and isoniazid twice a week for treatment of drug-susceptible pulmonary tuberculosis in HIV-negative patients: a randomised clinical trial. Lancet.

[8] Goldberg, S. TBTC Study 31: rifapentine-containing tuberculosis treatment shortening regimens (S31/A5349). ClinicalTrials.gov https://clinicaltrials.gov/ct2/show/NCT02410772 (2015).

[9] Johnson JL (2009). Shortening treatment in adults with noncavitary tuberculosis and 2-month culture conversion. Am. J. Respir. Crit. Care Med..

[10] Savic RM (2017). Defining the optimal dose of rifapentine for pulmonary tuberculosis: exposure-response relations from two phase II clinical trials. Clin. Pharmacol. Ther..

[11] Alipanah N (2016). Treatment of non-cavitary pulmonary tuberculosis with shortened fluoroquinolone-based regimens: a meta-analysis. Int. J. Tuberc. Lung Dis..

[12] Vernon, A. A. & Iademarco, M. F. In the treatment of tuberculosis, you get what you pay for... *Am. J. Respir. Crit. Care Med.* **170**, 1040–1042 (2004). 10.1164/rccm.2409005 15533952

[13] Chang KC, Leung CC, Yew WW, Ho SC, Tam CM (2004). A nested case–control study on treatment-related risk factors for early relapse of tuberculosis. Am. J. Respir. Crit. Care Med..

[14] Ngwatu Brian Kermu, Nsengiyumva Ntwali Placide, Oxlade Olivia, Mappin-Kasirer Benjamin, Nguyen Nhat Linh, Jaramillo Ernesto, Falzon Dennis, Schwartzman Kevin (2018). The impact of digital health technologies on tuberculosis treatment: a systematic review. European Respiratory Journal.

[15] DiStefano MJ, Schmidt H (2016). mHealth for tuberculosis treatment adherence: a framework to guide ethical planning, implementation, and evaluation. Glob. Health Sci. Pract..

[16] Fox W, Ellard GA, Mitchison DA (1999). Studies on the treatment of tuberculosis undertaken by the British Medical Research Council tuberculosis units, 1946–1986, with relevant subsequent publications. Int. J. Tuberc. Lung Dis..

[17] Fox W (1981). Whither short-course chemotherapy?. Br. J. Dis. Chest.

[18] Kendall EA (2017). Priority-setting for novel drug regimens to treat tuberculosis: an epidemiologic model. PLoS Med..

[19] Clinical Data Interchange Standards Consortium. *CDISC Therapeutic Area Data Standards User Guide for Tuberculosis* (Version 2.0). https://www.cdisc.org/standards/therapeutic-areas/tuberculosis (2016).

[20] Taichman DB (2016). Sharing clinical trial data—a proposal from the International Committee of Medical Journal Editors. N. Engl. J. Med..

[21] Wang R, Lagakos SW, Ware JH, Hunter DJ, Drazen JM (2007). Statistics in medicine—reporting of subgroup analyses in clinical trials. N. Engl. J. Med..

[22] Xie J, Liu C (2005). Adjusted Kaplan–Meier estimator and log-rank test with inverse probability of treatment weighting for survival data. Stat. Med..

