# Supplementary material for: A patient-level pooled analysis of treatment-shortening regimens for drug-susceptible pulmonary tuberculosis
Source: Nat Med. 2018 Nov 5;24(11):1708–15. doi: 10.1038/s41591-018-0224-2 (PMC6685538; doi:10.1038/s41591-018-0224-2)
Supplement: Supplementary file 1 — Supplementary Figures 1–3 and Supplementary Tables 1–12 [file 41591_2018_224_MOESM1_ESM.pdf]

SUPPLEMENTARY INFORMATION
https://doi.org/10.1038/s41591-018-0224-2

nature medicine

In the format provided by the authors and unedited.

A patient-level pooled analysis of treatment-shortening regimens for drug-susceptible pulmonary tuberculosis

Marjorie Z. Imperial[1,11], Payam Nahid[1,11], Patrick P. J. Phillips[1], Geraint R. Davies[2], Katherine Fielding[3], Debra Hanna[4,5], David Hermann[5], Robert S. Wallis[6], John L. Johnson[7,8], Christian Lienhardt[9,10] and Rada M. Savic[1]*

[1]University of California, San Francisco, San Francisco, CA, USA. [2]University of Liverpool, Liverpool, UK. [3]London School of Hygiene and Tropical Medicine, London, UK. [4]Critical Path Institute, Tucson, AZ, USA. [5]Bill and Melinda Gates Foundation, Seattle, WA, USA. [6]Aurum Institute and ACT4TB/HIV, Johannesburg, South Africa. [7]Case Western Reserve University, Cleveland, OH, USA. [8]University Hospitals Cleveland Medical Center, Cleveland, OH, USA. [9]Global Tuberculosis Programme, World Health Organization, Geneva, Switzerland. [10]Unité Mixte Internationale TransVIHMI (UMI 233 IRD–U1175 INSERM–Université de Montpellier), Institut de Recherche pour le Développement (IRD), Montpellier, France. [11]These authors contributed equally: Marjorie Z. Imperial, Payam Nahid. *e-mail: rada.savic@ucsf.edu

Supplementary Appendix

Supplement to: Imperial M, Nahid P, et al. A Patient-Level Pooled Analysis of Treatment Shortening Regimens for Drug-Susceptible Pulmonary Tuberculosis

Summary of Sensitivity/Additional Analyses

The following sensitivity and additional analyses were performed:

1. Multivariate Cox proportional hazard analysis in PP analysis population (Table S6)
2. Univariate Cox proportional hazard analysis in modified intent-to-treat analysis populations (Table S8)
3. Multivariate Cox proportional hazard analysis with cavitation status included *a priori* (Table S9 and Table S10).
4. Multivariate Cox proportional hazard analysis with inclusion of all mycobacterial interspersed repetitive unit (MIRU)-confirmed reinfections, classified as unfavorable (Table S11) or favorable, or completely removed from the analysis. Results were similar in all analyses.
5. Univariate Cox proportional hazard analysis for merged mycobacteria growth indicator tube system (MGIT) and Lowenstein-Jensen (LJ) culture data (as used in primary analysis), MGIT data only, and LJ data only. Results were similar in all analyses (Table S12).
6. Multivariate Cox proportional hazard analysis of impact of adherence with cut-off for total number of doses of at least 130 (exact number of doses if participant took 5/7 doses for 26 weeks) for the REMoxTB and RIFAQUIN analysis and 120 (exact number of doses if participant took 5/7 doses for 24 weeks) for the OFLOTUB analysis. Results and conclusions were similar when compared to the original analysis with a cutoff of at least 112 total doses.
7. Multivariate Cox proportional hazard analysis with events defined as TB related outcomes and all other outcomes censored (details on definition of TB related outcome in Table S1). Results were similar when compared to primary analysis in which events included all trial defined unfavorable outcomes.
8. Univariate Cox proportional hazard analysis with X-Ray Measurements (collection details available in Table S2; results available in Figure S1 and Figure S2)

Supplemental Figure 1. Univariate Cox proportional hazard analysis with X-Ray measurements in the MITT analysis population for the experimental group. Measurements collected at baseline are shown in the top panel and measurements collected at the end of treatment are shown in the bottom panel. Cavity size at baseline was collected in the RIFAQUIN trial. Disease Extent, Disease Grade, and Lung Zone Score was collected in the OFLOTUB trial. Hazard ratios with 95% Wald confidence interval are reported.

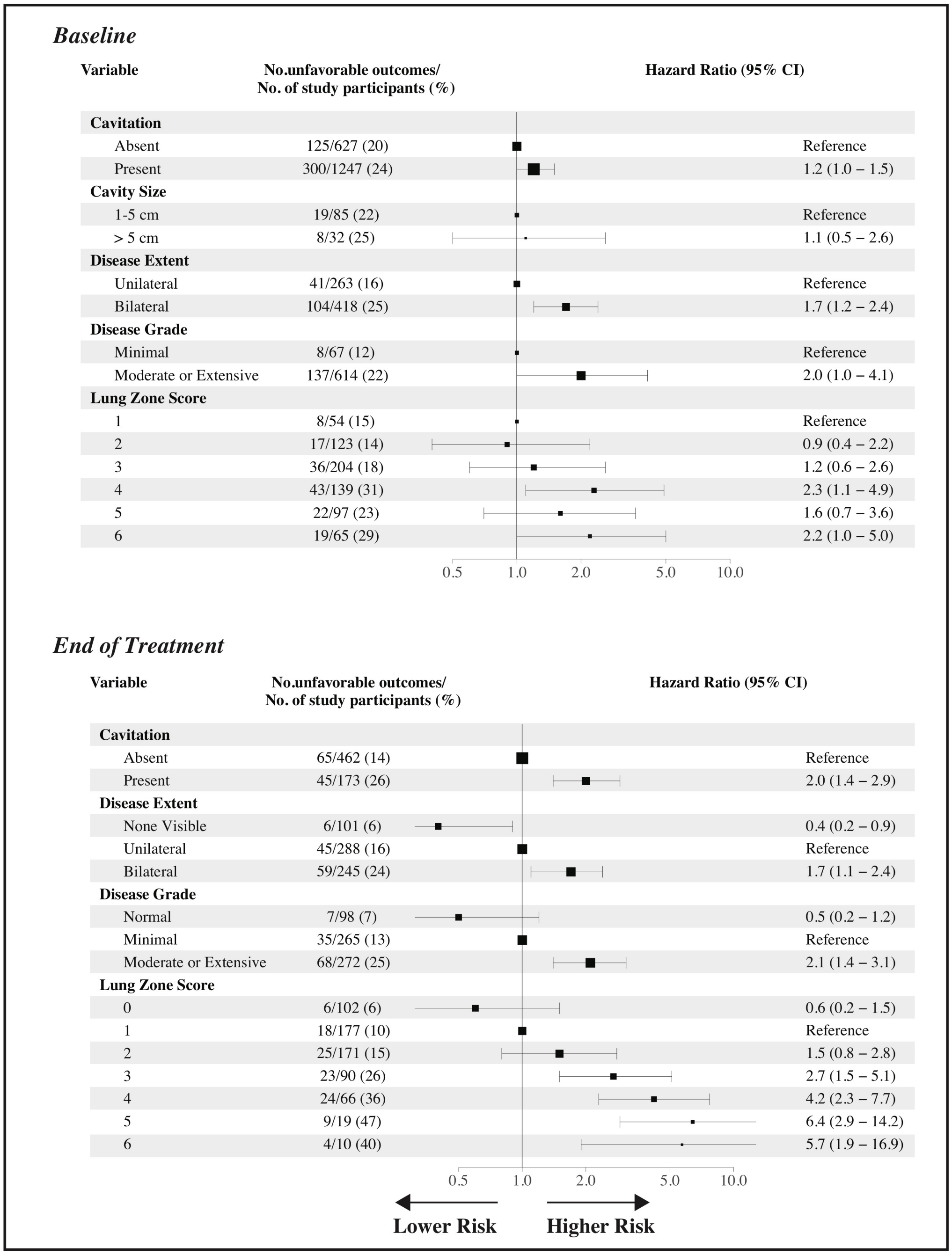

Supplemental Figure 2. Univariate Cox proportional hazard analysis with X-Ray measurements in the MITT analysis population for the control group. Measurements collected at baseline are shown in the top panel and measurements collected at the end of treatment are shown in the bottom panel. Cavity size at baseline was collected in the RIFAQUIN trial. Disease Extent, Disease Grade, and Lung Zone Score was collected in the OFLOTUB trial. Hazard ratios with 95% Wald confidence interval are reported.

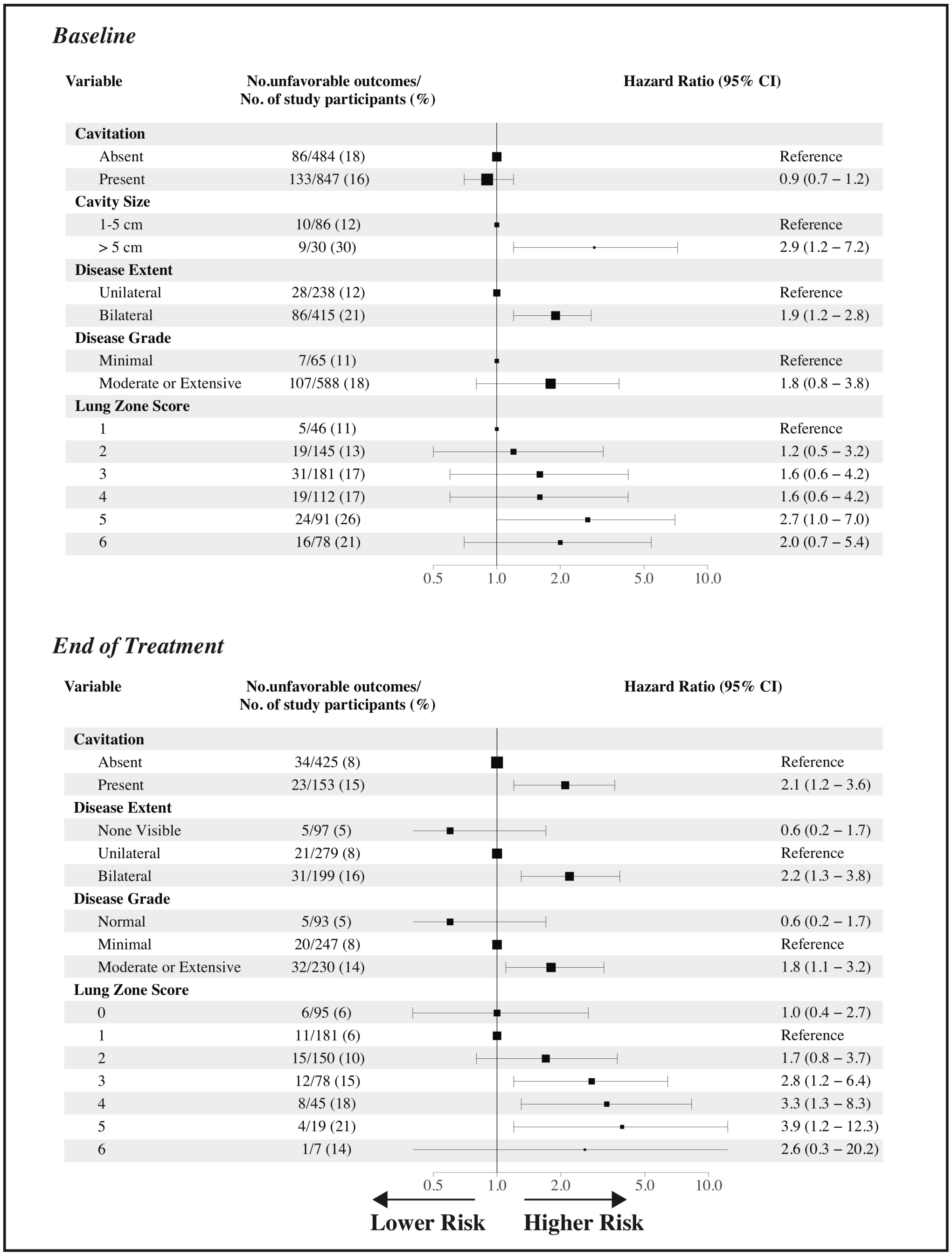

Supplementary Figure 3. Correlation matrix based on Spearman coefficient between variables of interest. In the top right half of the figure, a saturated blue box suggests a strong positive correlation (Spearman coefficients goes toward 1) and a saturated red box suggest a strong negative correlation (Spearman coefficients goes toward -1) between two variables. Bottom left half of the figure show Spearman coefficients between two variables. NA: Correlation not applicable.

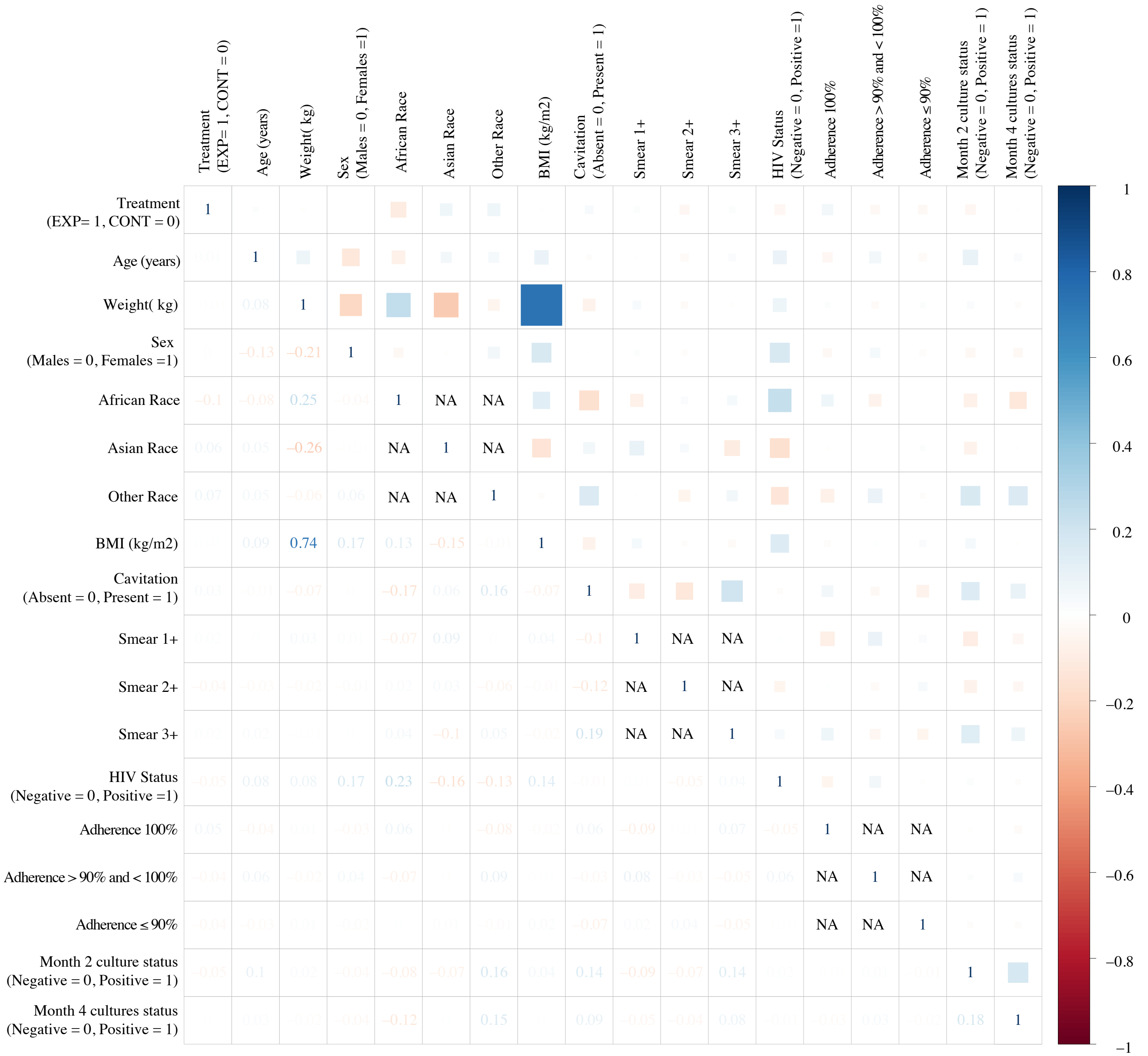

Supplemental Table 1. Definitions of favorable and unfavorable outcomes per clinical trial.

Outcomes defined as favorable are shown in green and outcomes defined as unfavorable are shown in red. Refer to trial protocols for exact criteria/definition for each outcome category. N refers to number of study participants in MITT analysis population** with corresponding outcome.

Trial	Outcome	N
**OFLOTUB**	Favorable	1090*
	By end of treatment, died	14
	By end of treatment, adverse event	2
	By end of treatment, drop-out	52
	By end of treatment, consent withdrawn	16
	By end of treatment, treatment failure[‖]	28
	After end of treatment, TB-recurrence, two positive culture[¶,‖]	119
	After end of treatment, TB-recurrence, one positive culture[¶,‖]	21
	After end of treatment, culture negative/unk[ʃ,‖]	8
**REMoxTB**	Culture-negative status at 18 mo.	1166
	Unable to produce sputum	2
	Unable to produce sputum at 18 mo. But culture negative status earlier	115
	Missing data on LJ culture at 18 mo. And MGIT negative	40
	6-mo. treatment phase[†], nonviolent death	18
	6 mo. treatment phase, adverse reaction	42
	6 mo. treatment phase, withdrawal of consent	34
	6 mo. treatment phase, relocation	10
	6 mo. treatment phase, other investigator decision	7
	6 mo. treatment phase, no completion of treatment	29
	6-mo. treatment phase, treatment failure, culture confirmed[‖]	8
	6 mo. treatment phase, treatment failure, not culture confirmed[‖]	9
	Follow up, relapse after culture negative status[‖]	123
	Follow up, retreated for tuberculosis[‖]	59
	Follow up, no culture negative status ever[‖]	3
	Follow up, no culture negative status at last visit[‖]	7
	Follow up, death from tuberculosis or respiratory distress[‖]	2
**RIFAQUIN**	Favorable	302
	During treatment, death	1
	During treatment, change in treatment due to adverse event	3
	During treatment, lost to follow up	11
	During treatment, inadequate treatment	3
	During treatment, other treatment change	21
	During treatment, failure (culture confirmed)[‖]	4
	After treatment, relapse, culture confirmation[‖]	23
	After treatment, relapse, limited bacteriologic confirmation[‖]	10
	After treatment, culture positive when last seen[‖]	2
	After treatment, death due to tuberculosis[‖]	1
**DMID 01-009****	Microbiological cure	326
	Clinical cure	28
	Bacteriological relapse[,‖]	18
	Death	4
	Lost to follow up	9
	Other	1

*Six study participants were excluded due inability to verify treatment allocation in source database
[†] In the REMoxTB trial, treatment phase was defined as 32 weeks after randomization.
[ʃ]smear positive/symptoms

¶Sixteen TB-recurrences included in the primary analysis for the OFLOTUB trial were MIRU confirmed reinfections
‖Included in TB related outcome definition.
**For DMID 01-009 trial, data corresponds to time to event analysis population from original publication.

Supplemental Table 2. Dataset specification and harmonization

Variable	Data Type and Format	Collected?				No. participants with missing data (%*)	Included in primary analysis?	Notes
		OFLOTUB	REMoxTB	RIFAQUIN	DMID01-009			
Study country	Categorical: 1-Benin, 2-Botswana 3-China, 4-Guinea, 5-India, 6-Kenya, 7-Mexico, 8-Malaysia, 9-Senegal, 10- Thailand, 11-Tanzania, 12-South Africa, 13-Zambia, 14-Zimbabwe, 101- Uganda, 102- Philippines, 103-Brazil	Yes	Yes	Yes	Yes	0 (0)	Yes	
Age	Number (units, years)	Yes	Yes	Yes	Yes	5 (<1)	Yes	
Sex	Boolean: 0-Female, 1- Male	Yes	Yes	Yes	Yes	0 (0)	Yes	
Race	Categorical: 1-Black, 2-Asian, 3-Other	No	Yes	Yes	No	1736 (46)	Yes	Black race was assigned to all study participants in OFLOTUB trial, given all OFLOTUB sites were in Africa. Race was not a significant risk factor in primary analysis, therefore, was not imputed for validation.

Weight	Number (units, kg)	Yes	Yes	Yes	Yes	0 (0)	No	Not included in primary analysis because of correlation with BMI (Figure S1)
Body mass index (BMI)	Number (units, kg/m^2)	Calculated - see notes	Calculated- see notes	Calculated - see notes	Calculated- see notes	291(8) with missing height-see notes	Yes	Body mass index was defined as the weight in kilograms divided by the squared height in meters. Height was not available for 291 study participants in the RIFAQUIN trial, median height from available data was assigned according to sex.
HIV status	Boolean: 0-negative, 1-positive	Yes	Yes	Yes	Yes	9 (<1)	Yes	According to original trial protocols, OFLOTUB trial excluded participants who required concomitant anti-infective treatment or HIV infected participants with WHO stage 3 infection (except those presenting with only the "loss of weight>10% body weight" criterion) and all participants at WHO stage 4, REMoxTB trial excluded HIV infected participants already receiving anti-retroviral therapy or CD4 count less than 250 cells/μL, RIFAQUIN trial excluded HIV infected participants who required anti-retroviral therapy at diagnosis (amended to “already receiving anti-

								retroviral therapy” during course of trial) or CD4 cell count less than 200 cells/μL (amended to 150 cells/μL during course of trial), and DMID 01-009 trial excluded all HIV infected participants.
Baseline CD4 cell count	Number (units, cells/μL)	Yes	Yes	Yes	NA	79 (17 †)	No	See notes for HIV status
Smoking	Categorical: 0-No, 1-Before, 2-Ongoing	No	Yes	Yes	No	1736 (46)	No	
Alcohol Use	Boolean: 0-No, 1-Yes	No	No	No	Yes	3405 (90)	No	
Drug Use	Boolean: 0-No, 1-Yes	No	No	No	Yes	3405 (90)	No	
Pharmacogenomics (Isoniazid acetylator)	NA	No	No	No	No	3791 (100)	No	
Baseline cough	Boolean: 0-No, 1-Yes	Yes	Yes	No	Yes	381 (10)	No	Cough grade data was available in REMoxTB trial. Cough data during treatment and follow-up available in DMID 01-009 trial.
Baseline smear grade ††	Categorical: 1-Smear negative or 1+, 2-Smear 2+, 3-Smear 3+	Yes	Yes	Yes	Yes	43 (1)	Yes	A conversion chart available in the REMoxTB trial lab manual was used to readjust all smear data to the same grading scale (Additional details in Supplementary Methods). Longitudinal data available in all trials.
Baseline cavitation ††	Boolean: 0-Absent,	Yes	Yes	Yes	Yes ∫	200 (5)	Yes	Follow-up data available in OFLOTUB trial.

	1-Present							
Baseline cavity size ††	Categorical: 0- 1-5 cm, 1- >5 cm	No	No	Yes	Yes ∫	1861 (89 ¶)	No	
Baseline lung disease grade ††	Categorical: 0-Normal, 1-Minimal, 2-Moderately Advanced, 3-Far Advanced	Yes	No	No	Yes	2057 (54)	No	Follow-up data available in OFLOTUB and DMID 01-009 trials.
Baseline lung disease extent ††	Categorical: 0-None Visible, 1-Unilateral 2-Bilateral	Yes	No	No	No	2458 (65)	No	Follow-up data available in OFLOTUB and DMID 01-009 trials.
Baseline lung zone score ††	Categorical: Factors 0 to 6; 0-no area affected, 6-all lung regions affected	Yes	No	No	No	2457 (65)	No	Follow up data available in OFLOTUB trial.
On-treatment culture status based on LJ medium ††	Boolean: 0-Negative 1-Positive	Yes	Yes	Yes ǁ	Yes	Month 2: 524 (14) Month 4: 348 (19**)	No	Longitudinal data available in all trials.
On-treatment culture status based on MGIT medium ††	Boolean: 0-Negative 1-Positive	No	Yes	Yes ǁ	No	Month 2: 2094 (55) Month 4: 1187 (66**)	No	Longitudinal data available in all trials
On-treatment culture status based on LJ or MGIT medium (data used in primary analysis) ††	Boolean: 0-Negative 1-Positive	Yes	Yes	Yes	Yes	Month 2: 308 (8) Month 4: 236 (13**)	Month 2- Yes Month 4- No	Longitudinal data available in all trials
Drug regimen	Categorical: See description of	Yes	Yes	Yes	Yes	6 (<1)	No	Six study participants were excluded from the current analyses due to

	study arms in original protocols, methods, and supplementary methods							untraceable regimen assignment in the source database.
Drug Dose	Number: See description in original protocols	Yes	Yes	Yes	Yes	6 (<1)	No	Six study participants were excluded from the current analyses due inability to verify treatment allocation in source database.
Treatment Adherence	Number (units, %)	Calculated - see notes	Calculated- see notes	Calculated - see notes	No	83 (2 ∫ ∫)	Yes	Treatment adherence was calculated as the number of days that doses were taken divided by the prescribed number of days.
Pharmacokinetics	Number: See description in original protocols	Sub-study	No	Sub-study	No	3207 (86)	No	
Drug Resistance	Categorical: See description in original protocols	Yes	Yes	Yes	Yes	Variable- based on exclusion/inclusion criteria- see notes	No	According to original trial protocols, OFLOTUB trial withdrew participants resistant to rifampin, REMoxTB trial excluded participants resistant to rifampin or any fluoroquinolone, RIFAQUIN trial excluded individuals resistant to rifampin, isoniazid, or moxifloxacin, and DMID 01-009 trial, excluded individuals resistant to rifampin, isoniazid, ethambutol or pyrazinamide.

* Percentage based on entire population, which included 3405 participants from REMoxTB, RIFAQUIN, OFLOTUB and 386 from DMID 01-009 studies.
† Percentage based on HIV infected population.
∫ DMID 01-009 study population were non-cavitary disease adults.
¶ Percentage based on study participants with cavitary disease.
‖ Study site specific.
** Percentage based on 6-month control population only.
†† Worst outcomes used for analysis if multiple samples available.
∫ ∫ Missing population and percentage based on REMoxTB, RIFAQUIN, OFLOTUB datasets only.
NA- not applicable

Supplemental Table 3. Summary of analysis populations with complete records of predefined predictors.

		Total No. of participants	No. of participants with complete records of baseline predictors (% of total)*	No. Participants with complete records of baseline and on-treatment predictors (% of total)*
**Experimental Group**	**MITT**	2001	1843 (92)	1668 (83)
	**PP**	1851	1711 (92)	1615 (87)
**Control Group**	**MITT**	1404	1311 (93)	1186 (84)
	**PP**	1271	1189 (94)	1127 (89)

*Baseline predictors include age, race, BMI, sex, presence of cavitation and smear grade. On-treatment predictors included adherence and month 2 culture status. Complete record defined after (1) black race was assigned for all participants in the OFLOTUB trial, in which race information was not available, given that all OFLTOUB sites were in Africa and similar demographic characteristics were observed in other studies at their African sites (majority black) and (2) median height for females and males of available data was used for 291 participants with missing height to calculate BMI.

Supplemental Table 4. Multivariate Cox proportional hazard analysis in MITT analysis population for experimental group. Multivariate results for **a.** baseline predictors and **b.** baseline and on-treatment predictors.

a.

Variable	N unfavorable/ N assessable (%)	Hazard Ratio (95% CI)	P value
Female	90/549 (16)	Reference	Reference
Male	326/1294 (25)	1.6 (1.3 - 2.1)	<0.001
Smear neg. or 1+	81/443 (18)	Reference	Reference
Smear 2+	104/481 (22)	1.2 (0.9 - 1.7)	0.17
Smear 3+	231/919 (25)	1.4 (1.1 - 1.9)	0.007
HIV negative	352/1615 (22)	Reference	Reference
HIV positive	64/228 (28)	1.4 (1.1 - 1.9)	0.01
Age (per 10 year increase)	< 30 years, 179/916 (20%)<br>≥ 30 years, 237/927 (26%)	1.1 (1.0 - 1.2)	0.02
South Africa	192/770 (25)	Reference	Reference
Benin	29/120 (24)	0.9 (0.6 - 1.4)	0.71
Botswana	2/11 (18)	0.8 (0.2 - 3.1)	0.71
China	3/12 (25)	1.3 (0.4 - 4.2)	0.63
Guinea	24/188 (13)	0.5 (0.3 - 0.8)	0.002
India	45/182 (25)	1.1 (0.8 - 1.5)	0.68
Kenya	30/151 (20)	0.8 (0.5 - 1.1)	0.16
Malaysia	17/43 (40)	2.0 (1.2 - 3.3)	0.008
Senegal	29/127 (23)	0.8 (0.6 - 1.2)	0.34
Thailand	7/65 (11)	0.4 (0.2 - 0.9)	0.02
Tanzania	17/91 (19)	0.8 (0.5 - 1.2)	0.27
Zambia	9/26 (35)	1.3 (0.7 - 2.6)	0.42
Zimbabwe	12/57 (21)	0.8 (0.4 - 1.4)	0.35

b.

Variable	N unfavorable/ N assessable (%)	Hazard Ratio (95% CI)	P value
Adherence 100%	238/1348 (18)	Reference	Reference
Adherence > 90% and <100%	64/288 (22)	1.4 (1.0 - 1.9)	0.03
Adherence ≤ 90%	15/32 (47)	5.7 (3.3 - 9.9)	<0.001
Month 2 culture negative	212/1357 (16)	Reference	Reference
Month 2 culture positive	105/311 (34)	2.2 (1.7 - 2.9)	<0.001
Females	64/492 (13)	Reference	Reference
Males	253/1176 (22)	1.6 (1.2 - 2.1)	<0.001
Smear neg. or 1+	53/388 (14)	Reference	Reference
Smear 2+	72/430 (17)	1.2 (0.8 - 1.7)	0.41
Smear 3+	192/850 (23)	1.6 (1.2 - 2.3)	0.002

HIV negative	270/1463 (18)	Reference	Reference
HIV positive	47/205 (23)	1.5 (1.1 - 2.0)	0.02
BMI (per 5 kg/m² decrease)	≥ 17 kg/m2, 226/1247 (18%)<br>< 17 kg/m2, 91/421 (22%)	1.4 (1.1 - 1.7)	0.007
Age (per 10 year increase)	< 30 years, 136/830 (16%)<br>≥ 30 years, 181/838 (22%)	1.1 (1.0 - 1.2)	0.04
South Africa	150/702 (21)	Reference	Reference
Benin	19/107 (18)	0.9 (0.5 - 1.4)	0.53
Botswana	2/11 (18)	1.2 (0.3 - 4.8)	0.81
China	2/10 (20)	1.4 (0.3 - 5.7)	0.64
Guinea	17/174 (10)	0.5 (0.3 - 0.8)	0.003
India	25/129 (19)	1.0 (0.6 - 1.6)	1.0
Kenya	26/145 (18)	1.0 (0.6 - 1.5)	0.84
Malaysia	13/39 (33)	2.4 (1.3 - 4.4)	0.003
Senegal	26/123 (21)	1.0 (0.6 - 1.6)	0.96
Thailand	6/64 (9)	0.6 (0.3 - 1.3)	0.2
Tanzania	12/84 (14)	0.7 (0.4 - 1.3)	0.24
Zambia	9/25 (36)	1.8 (0.9 - 3.6)	0.09
Zimbabwe	10/55 (18)	0.8 (0.4 - 1.5)	0.5

Supplemental Table 5. Multivariate Cox proportional hazard analysis in MITT analysis population for control group. Multivariate results for **a.** baseline predictors and **b.** baseline and on-treatment predictors.

a.

Variable	N unfavorable/ N assessable (%)	Hazard Ratio (95% CI)	P value
HIV negative	159/1103 (14)	Reference	Reference
HIV positive	54/208 (26)	2.3 (1.6 - 3.3)	<0.001
Female	47/381 (12)	Reference	Reference
Male	166/930 (18)	1.5 (1.1 - 2.1)	0.03
Age (per 10 year increase)	< 30 years, 92/657 (14%)<br>≥ 30 years, 121/654 (19%)	1.3 (1.1 - 1.4)	<0.001
BMI (per 5 kg/m^2 decrease)	≥ 17 kg/m2, 156/989 (16%)<br>< 17 kg/m2, 57/322 (18%)	1.3 (1.0 - 1.7)	0.04
South Africa	80/491 (16)	Reference	Reference
Benin	25/106 (24)	1.4 (0.9 - 2.2)	0.17
Botswana	0/11 (0)	NA	NA
China	0/8 (0)	NA	NA
Guinea	32/179 (18)	1.2 (0.8 - 1.9)	0.36
India	18/93 (19)	1.5 (0.9 - 2.5)	0.16
Kenya	18/115 (16)	1.1 (0.6 - 1.8)	0.8
Malaysia	5/19 (26)	2.4 (1.0 - 6.0)	0.06
Senegal	16/137 (12)	0.7 (0.4 - 1.3)	0.31
Thailand	5/34 (15)	1.0 (0.4 - 2.5)	0.99
Tanzania	4/53 (8)	0.5 (0.2 - 1.4)	0.18
Zambia	1/18 (6)	0.3 (0.0 - 2.3)	0.25
Zimbabwe	9/47 (19)	1.2 (0.6 - 2.5)	0.53

b.

Variable	N unfavorable/ N assessable (%)	Hazard Ratio (95% CI)	P value
Adherence 100%	85/913 (9)	Reference	Reference
Adherence > 90% and <100%	37/230 (16)	2.4 (1.6 - 3.6)	<0.001
Adherence ≤ 90%	16/43 (37)	5.9 (3.3 - 10.5)	<0.001
HIV negative	98/999 (10)	Reference	Reference
HIV positive	40/187 (21)	3.1 (2.0 - 4.6)	<0.001
Month 2 culture negative	93/922 (10)	Reference	Reference
Month 2 culture positive	45/264 (17)	1.8 (1.3 - 2.7)	0.002
BMI (per 5 kg/m^2 decrease)	≥ 17 kg/m2, 102/901 (11%)<br>< 17 kg/m2, 36/285 (13%)	1.5 (1.0 - 2.0)	0.03

Females	30/347 (9)	Reference	Reference
Males	108/839 (13)	1.5 (1.0 - 2.4)	0.05
South Africa	57/454 (13)	Reference	Reference
Benin	13/90 (14)	1.4 (0.7 - 2.7)	0.3
Botswana	0/7 (0)	NA	NA
China	0/7 (0)	NA	NA
Guinea	21/164 (13)	1.0 (0.6 - 1.7)	1
India	7/68 (10)	1.4 (0.6 - 3.1)	0.46
Kenya	14/110 (13)	1.6 (0.9 - 2.9)	0.14
Malaysia	3/17 (18)	2.9 (0.9 - 9.4)	0.08
Senegal	12/128 (9)	1.1 (0.6 - 2.2)	0.74
Thailand	2/31 (6)	1.0 (0.2 - 4.1)	0.98
Tanzania	4/52 (8)	0.8 (0.3 - 2.2)	0.63
Zambia	1/18 (6)	0.6 (0.1 - 4.4)	0.61
Zimbabwe	4/40 (10)	0.4 (0.2 - 1.3)	0.13

Supplemental Table 6. Multivariate Cox proportional hazard analysis in PP analysis population. Multivariate results with baseline and on-treatment predictors for **a.** experimental group study participants and **b.** control group study participants.

a.

Variable	N unfavorable/ N assessable (%)	Hazard Ratio (95% CI)	P value
Month 2 culture negative	182/1315 (14)	Reference	Reference
Month 2 culture positive	94/300 (31)	2.4 (1.9 - 3.2)	<0.001
Female	53/477 (11)	Reference	Reference
Male	223/1138 (20)	1.8 (1.3 - 2.4)	<0.001
Smear neg. or 1+	46/376 (12)	Reference	Reference
Smear 2+	59/414 (14)	1.1 (0.8 - 1.7)	0.59
Smear 3+	171/825 (21)	1.6 (1.1 - 2.3)	0.006
HIV negative	233/1415 (16)	Reference	Reference
HIV positive	43/200 (22)	1.6 (1.1 - 2.2)	0.008
BMI (per 5 kg/m^2 decrease)	≥ 17 kg/m^2: 196/1207 (16)<br>< 17 kg/m^2: 80/408 (20)	1.4 (1.1 - 1.8)	0.004
South Africa	132/680 (19)	Reference	Reference
Benin	14/100 (14)	0.8 (0.5 - 1.5)	0.5
Botswana	2/11 (18)	1.5 (0.4 - 6.0)	0.59
China	1/9 (11)	1.0 (0.1 - 7.0)	0.97
Guinea	12/164 (7)	0.4 (0.2 - 0.8)	0.006
India	24/128 (19)	1.1 (0.7 - 1.7)	0.7
Kenya	23/142 (16)	0.9 (0.5 - 1.3)	0.49
Malaysia	11/37 (30)	2.7 (1.4 - 5.1)	0.003
Senegal	26/122 (21)	1.1 (0.7 - 1.8)	0.59
Thailand	4/62 (6)	0.5 (0.2 - 1.3)	0.16
Tanzania	8/80 (10)	0.5 (0.2 - 1.0)	0.06
Zambia	9/25 (36)	1.8 (0.9 - 3.6)	0.09
Zimbabwe	10/55 (18)	1.1 (0.6 - 2.1)	0.83

b.

Variable	N unfavorable/ N assessable (%)	Hazard Ratio (95% CI)	P value
Adherence 100%	73/901 (8)	Reference	Reference
Adherence >90% and <100%	21/208 (10)	1.8 (1.1 - 3.0)	0.02
Adherence ≤ 90%	4/18 (22)	3.8 (1.3 - 10.7)	0.01
HIV negative	71/956 (7)	Reference	Reference
HIV positive	27/171 (16)	3.3 (2.0 - 5.5)	<0.001
Month 2 culture negative	61/874 (7)	Reference	Reference
Month 2 culture positive	37/253 (15)	2.5 (1.6 - 3.9)	<0.001

Female	18/332 (5)	Reference	Reference
Male	80/795 (10)	2.2 (1.3 - 3.8)	0.003
South Africa	42/437 (10)	Reference	Reference
Benin	5/80 (6)	0.9 (0.3 - 2.4)	0.87
Botswana	0/7 (0)	NA	NA
China	0/7 (0)	NA	NA
Guinea	15/146 (10)	1.8 (0.9 - 3.5)	0.09
India	6/67 (9)	2.0 (0.8 - 4.9)	0.13
Kenya	11/107 (10)	1.7 (0.9 - 3.5)	0.13
Malaysia	2/16 (12)	2.7 (0.6 - 11.6)	0.17
Senegal	11/126 (9)	1.6 (0.7 - 3.4)	0.23
Thailand	1/30 (3)	0.7 (0.1 - 5.1)	0.72
Tanzania	3/51 (6)	0.8 (0.3 - 2.7)	0.74
Zambia	1/18 (6)	0.8 (0.1 - 5.6)	0.78
Zimbabwe	1/35 (3)	0.3 (0.0 - 2.0)	0.2

Supplementary Table 7. Analysis of 7/7 and 6/7 dosing strategies and impact of adherence in the control group. Multivariate analysis with total number of doses taken for study participants who took at least 4 months of treatment under 7/7 dosing strategies for 26 weeks (REMoxTB and RIFAQUIN trials). **b.** Multivariate analysis with total number of doses taken for study participants who took at least 4 months of treatment under 6/7 dosing strategies for 24 weeks (OFLOTUB trial).

a.

Variable	N unfavorable/ N assessable (%)	Hazard Ratio (95% CI)	P value
182 (7 doses per week)	26/452 (6)	Reference	Reference
156-181 (on average 6 doses per week)	22/217 (10)	2.4 (1.3 - 4.3)	0.004
112-155 (on average 5 doses per week)	13/18 (72)	28.9 (10.5 - 80.0)	<0.001
Treatment duration (per week)	< 182 days, 21/110 (19%)<br>≥ 182 days, 40/577 (7%)	0.9 (0.8 - 1.1)	0.25
South Africa	37/342 (11)	Reference	Reference
Botswana	0/12 (0)	NA	NA
China	0/8 (0)	NA	NA
India	11/102 (11)	1.6 (0.8 - 3.1)	0.22
Kenya	0/37 (0)	NA	NA
Malaysia	3/18 (17)	1.7 (0.5 - 5.5)	0.41
Thailand	2/31 (6)	1.0 (0.2 - 4.1)	0.96
Tanzania	4/65 (6)	0.8 (0.3 - 2.3)	0.7
Zambia	1/21 (5)	0.8 (0.1 - 5.7)	0.79
Zimbabwe	3/51 (6)	0.2 (0.0 - 0.6)	0.004

b.

Variable	N unfavorable/ N assessable (%)	Hazard Ratio (95% CI)	P value
144 (6 doses per week)	50/533 (9)	Reference	Reference
112-143 (on average 5 doses per week)	13/65 (20)	2.4 (1.2 - 4.8)	0.02
Treatment duration (per week)	< 169 days, 21/155 (14%)<br>≥ 169 days, 42/443 (9%)	0.7 (0.5 - 0.9)	0.002
South Africa	21/146 (14)	Reference	Reference
Benin	4/86 (5)	0.3 (0.1 - 0.8)	0.01
Guinea	17/163 (10)	0.4 (0.2 - 0.7)	0.004
Kenya	10/76 (13)	0.9 (0.4 - 1.9)	0.74
Senegal	11/127 (9)	0.5 (0.2 - 1.1)	0.07
Zambia	1/21 (5)	0.8 (0.1 - 5.7)	0.79
Zimbabwe	3/51 (6)	0.2 (0.0 - 0.6)	0.004

Supplementary Table 8. Summary of univariate Cox proportional hazard analysis in the MITT analysis population. Univariate results for **a.** experimental group study participants and **b.** control group study participants. Unadjusted analysis.

a.

Variable	N unfavorable/ N assessable (%)	Hazard Ratio (95% CI)	P value
Age (per 10 year increase)	< 30 years: 195/990 (20)<br>≥ 30 years: 265/1007 (26)	1.1 (1.0 - 1.2)	0.002
BMI (per 5 kg/m² decrease)	≥ 17 kg/m²: 320/1472 (22)<br>< 17 kg/m²: 142/529 (27)	1.2 (1.0 - 1.4)	0.08
Cavitation absent	125/627 (20)	Reference	Reference
Cavitation present	300/1247 (24)	1.2 (1.0 - 1.5)	0.05
HIV negative	387/1748 (22)	Reference	Reference
HIV positive	73/248 (29)	1.4 (1.1 - 1.8)	0.009
Female	100/592 (17)	Reference	Reference
Male	362/1409 (26)	1.6 (1.3 - 2.0)	<0.001
Adherence 100%	313/1572 (20)	Reference	Reference
Adherence > 90% and <100%	77/335 (23)	1.2 (0.9 - 1.5)	0.14
Adherence ≤ 90%	32/54 (59)	4.8 (3.3 - 6.9)	<0.001
African race	294/1326 (22)	Reference	Reference
Asian race	90/349 (26)	1.2 (1.0 - 1.6)	0.08
Other race	78/326 (24)	1.1 (0.9 - 1.4)	0.44
Smear neg. or 1+	88/483 (18)	Reference	Reference
Smear 2+	108/503 (21)	1.2 (0.9 - 1.6)	0.24
Smear 3+	259/988 (26)	1.5 (1.2 - 1.9)	0.001
Month 2 culture negative	248/1478 (17)	Reference	Reference
Month 2 culture positive	115/336 (34)	2.3 (1.9 - 2.9)	<0.001

b.

Variable	N unfavorable/ N assessable (%)	Hazard Ratio (95% CI)	P value

Age (per 10 year increase)	< 30 years: 98/708 (14)<br>≥ 30 years: 130/695 (19)	1.3 (1.1 - 1.4)	<0.001
BMI (per 5 kg/m² decrease)	≥ 17 kg/m²: 166/1052 (16)<br>< 17 kg/m²: 62/352 (18)	1.2 (1.0 - 1.6)	0.08
Cavitation absent	86/484 (18)	Reference	Reference
Cavitation present	133/847 (16)	0.9 (0.7 - 1.2)	0.48
HIV negative	171/1180 (14)	Reference	Reference
HIV positive	56/220 (25)	1.9 (1.4 - 2.5)	<0.001
Female	56/415 (13)	Reference	Reference
Male	172/989 (17)	1.3 (1.0 - 1.8)	0.07
Adherence 100%	114/1031 (11)	Reference	Reference
Adherence > 90% and <100%	48/274 (18)	1.8 (1.3 - 2.5)	<0.001
Adherence ≤ 90%	25/56 (45)	5.1 (3.3 - 7.9)	<0.001
African race	169/1066 (16)	Reference	Reference
Asian race	33/178 (19)	1.4 (0.9 - 2.0)	0.1
Other race	26/160 (16)	1.2 (0.8 - 1.8)	0.48
Smear neg. or 1+	53/317 (17)	Reference	Reference
Smear 2+	56/404 (14)	0.8 (0.5 - 1.1)	0.22
Smear 3+	114/667 (17)	1.0 (0.7 - 1.4)	0.94
Month 2 culture negative	113/998 (11)	Reference	Reference
Month 2 culture positive	49/285 (17)	1.6 (1.1 - 2.2)	0.007

Supplemental Table 9. Multivariate Cox proportional hazard analysis in MITT analysis population for experimental group after inclusion of cavitation status *a priori*. Multivariate results for **a.** baseline predictors and **b.** baseline and on-treatment predictors.

a.

Variable	N unfavorable/ N assessable (%)	Hazard Ratio (95% CI)	P value
Female	90/549 (16)	Reference	Reference
Male	326/1294 (25)	1.6 (1.3 - 2.1)	<0.001
Smear neg. or 1+	81/443 (18)	Reference	Reference
Smear 2+	104/481 (22)	1.2 (0.9 - 1.7)	0.18
Smear 3+	231/919 (25)	1.4 (1.1 - 1.9)	0.009
HIV negative	352/1615 (22)	Reference	Reference
HIV positive	64/228 (28)	1.4 (1.1 - 1.9)	0.01
Age (per 10 year increase)	< 30 years, 179/916 (20%)<br>≥ 30 years, 237/927 (26%)	1.1 (1.0 - 1.2)	0.01
Cavitation absent	122/617 (20)	Reference	Reference
Cavitation present	294/1226 (24)	1.0 (0.8 - 1.3)	0.75
South Africa	192/770 (25)	Reference	Reference
Benin	29/120 (24)	1.0 (0.6 - 1.5)	0.82
Botswana	2/11 (18)	0.8 (0.2 - 3.1)	0.71
China	3/12 (25)	1.3 (0.4 - 4.2)	0.62
Guinea	24/188 (13)	0.5 (0.3 - 0.8)	0.004
India	45/182 (25)	1.1 (0.8 - 1.5)	0.67
Kenya	30/151 (20)	0.8 (0.5 - 1.2)	0.2
Malaysia	17/43 (40)	2.0 (1.2 - 3.3)	0.008
Senegal	29/127 (23)	0.8 (0.6 - 1.2)	0.34
Thailand	7/65 (11)	0.4 (0.2 - 0.9)	0.02
Tanzania	17/91 (19)	0.8 (0.5 - 1.2)	0.27
Zambia	9/26 (35)	1.3 (0.7 - 2.6)	0.41
Zimbabwe	12/57 (21)	0.8 (0.4 - 1.4)	0.37

b.

Variable	N unfavorable/ N assessable (%)	Hazard Ratio (95% CI)	P value
Adherence 100%	238/1348 (18)	Reference	Reference
Adherence > 90% and <100%	64/288 (22)	1.4 (1.0 - 1.9)	0.02
Adherence ≤ 90%	15/32 (47)	5.7 (3.3 - 9.9)	<0.001
Month 2 culture negative	212/1357 (16)	Reference	Reference
Month 2 culture positive	105/311 (34)	2.2 (1.7 - 2.9)	<0.001
Females	64/492 (13)	Reference	Reference
Males	253/1176 (22)	1.6 (1.2 - 2.1)	<0.001
Smear neg. or 1+	53/388 (14)	Reference	Reference

Smear 2+	72/430 (17)	1.2 (0.8 - 1.7)	0.42
Smear 3+	192/850 (23)	1.6 (1.2 - 2.3)	0.002
HIV negative	270/1463 (18)	Reference	Reference
HIV positive	47/205 (23)	1.5 (1.1 - 2.0)	0.02
BMI (per 5 kg/m^2 decrease)	≥ 17 kg/m2, 226/1247 (18%)<br>< 17 kg/m2, 91/421 (22%)	1.4 (1.1 - 1.7)	0.007
Age (per 10 year increase)	< 30 years, 136/830 (16%)<br>≥ 30 years, 181/838 (22%)	1.1 (1.0 - 1.2)	0.04
Cavitation absent	86/556 (15)	Reference	Reference
Cavitation present	231/1112 (21)	1.0 (0.8 - 1.4)	0.85
South Africa	150/702 (21)	Reference	Reference
Benin	19/107 (18)	0.9 (0.5 - 1.5)	0.61
Botswana	2/11 (18)	1.2 (0.3 - 4.8)	0.81
China	2/10 (20)	1.4 (0.3 - 5.8)	0.64
Guinea	17/174 (10)	0.5 (0.3 - 0.8)	0.006
India	25/129 (19)	1.0 (0.6 - 1.6)	0.99
Kenya	26/145 (18)	1.0 (0.6 - 1.5)	0.89
Malaysia	13/39 (33)	2.4 (1.3 - 4.4)	0.003
Senegal	26/123 (21)	1.0 (0.6 - 1.6)	0.96
Thailand	6/64 (9)	0.6 (0.3 - 1.3)	0.2
Tanzania	12/84 (14)	0.7 (0.4 - 1.3)	0.24
Zambia	9/25 (36)	1.8 (0.9 - 3.6)	0.09
Zimbabwe	10/55 (18)	0.8 (0.4 - 1.5)	0.51

Supplemental Table 10. Multivariate Cox proportional hazard analysis in MITT analysis population for control group after inclusion of cavitation status *a priori*. Multivariate results for **a.** baseline predictors and **b.** baseline and on-treatment predictors.

a.

Variable	N unfavorable/ N assessable (%)	Hazard Ratio (95% CI)	P value
HIV negative	159/1103 (14)	Reference	Reference
HIV positive	54/208 (26)	2.3 (1.7 - 3.3)	<0.001
Female	47/381 (12)	Reference	Reference
Male	166/930 (18)	1.5 (1.0 - 2.1)	0.03
Age (per 10 year increase)	< 30 years, 92/657 (14%)<br>≥ 30 years, 121/654 (19%)	1.3 (1.1 - 1.4)	<0.001
BMI (per 5 kg/m^2 decrease)	≥ 17 kg/m2, 156/989 (16%)<br>< 17 kg/m2, 57/322 (18%)	1.3 (1.0 - 1.7)	0.05
Cavitation absent	84/477 (18)	Reference	Reference
Cavitation present	129/834 (15)	1.1 (0.8 - 1.5)	0.67
South Africa	80/491 (16)	Reference	Reference
Benin	25/106 (24)	1.5 (0.9 - 2.4)	0.16
Botswana	0/11 (0)	NA	NA
China	0/8 (0)	NA	NA
Guinea	32/179 (18)	1.3 (0.8 - 2.1)	0.31
India	18/93 (19)	1.5 (0.9 - 2.6)	0.15
Kenya	18/115 (16)	1.1 (0.6 - 1.9)	0.7
Malaysia	5/19 (26)	2.4 (1.0 - 6.1)	0.06
Senegal	16/137 (12)	0.7 (0.4 - 1.3)	0.31
Thailand	5/34 (15)	1.0 (0.4 - 2.5)	1
Tanzania	4/53 (8)	0.5 (0.2 - 1.4)	0.19
Zambia	1/18 (6)	0.3 (0.0 - 2.3)	0.26
Zimbabwe	9/47 (19)	1.3 (0.6 - 2.6)	0.48

b.

Variable	N unfavorable/ N assessable (%)	Hazard Ratio (95% CI)	P value
Adherence 100%	85/913 (9)	Reference	Reference
Adherence > 90% and <100%	37/230 (16)	2.4 (1.6 - 3.6)	<0.001
Adherence ≤ 90%	16/43 (37)	6.1 (3.4 - 11.0)	<0.001
HIV negative	98/999 (10)	Reference	Reference
HIV positive	40/187 (21)	3.2 (2.1 - 4.9)	<0.001
Month 2 culture negative	93/922 (10)	Reference	Reference
Month 2 culture positive	45/264 (17)	1.8 (1.2 - 2.7)	0.002

BMI (per 5 kg/m² decrease)	≥ 17 kg/m2, 102/901 (11%)<br>< 17 kg/m2, 36/285 (13%)	1.4 (1.0 - 2.0)	0.05
Females	30/347 (9)	Reference	Reference
Males	108/839 (13)	1.5 (1.0 - 2.4)	0.05
Cavitation absent	47/430 (11)	Reference	Reference
Cavitation present	91/756 (12)	1.5 (1.0 - 2.4)	0.06
South Africa	57/454 (13)	Reference	Reference
Benin	13/90 (14)	1.9 (0.9 - 3.9)	0.08
Botswana	0/7 (0)	NA	NA
China	0/7 (0)	NA	NA
Guinea	21/164 (13)	1.3 (0.7 - 2.4)	0.39
India	7/68 (10)	1.4 (0.6 - 3.3)	0.39
Kenya	14/110 (13)	2.0 (1.0 - 3.9)	0.04
Malaysia	3/17 (18)	3.2 (1.0 - 10.6)	0.05
Senegal	12/128 (9)	1.2 (0.6 - 2.3)	0.68
Thailand	2/31 (6)	1.0 (0.2 - 4.3)	0.97
Tanzania	4/52 (8)	0.8 (0.3 - 2.3)	0.7
Zambia	1/18 (6)	0.7 (0.1 - 5.1)	0.72
Zimbabwe	4/40 (10)	0.5 (0.2 - 1.5)	0.22

Supplemental Table 11. Multivariate Cox proportional hazard analysis with all confirmed reinfections classified as an unfavorable outcome. Multivariate results with baseline and on-treatment predictors for **a.** experimental group study participants and **b.** control group study participants. Similar results were observed when reinfections were classified as favorable or completely removed from the analysis.

a.

Variable	N unfavorable/ N assessable (%)	Hazard Ratio (95% CI)	P value
Adherence 100%	256/1366 (19)	Reference	Reference
Adherence >90% and <100%	75/299 (25)	1.5 (1.2 - 2.0)	0.003
Adherence ≤ 90%	15/32 (47)	5.6 (3.2 - 9.6)	<0.001
Month 2 culture negative	232/1377 (17)	Reference	Reference
Month 2 culture positive	114/320 (36)	2.2 (1.7 - 2.8)	<0.001
Smear neg. or 1+	61/396 (15)	Reference	Reference
Smear 2+	78/436 (18)	1.1 (0.8 - 1.6)	0.52
Smear 3+	207/865 (24)	1.6 (1.2 - 2.1)	0.003
Female	72/500 (14)	Reference	Reference
Male	274/1197 (23)	1.5 (1.2 - 2.0)	0.002
BMI (per 5 kg/m² decrease)	≥ 17 kg/m²: 246/1267 (19)<br>< 17 kg/m²: 100/430 (23)	1.3 (1.1 - 1.6)	0.01
Age (per 10 year increase)	< 30 years: 150/844 (18)<br>≥ 30 years: 196/853 (23)	1.1 (1.0 - 1.2)	0.03
South Africa	160/712 (22)	Reference	Reference
Benin	19/107 (18)	0.8 (0.5 - 1.3)	0.36
Botswana	2/11 (18)	1.1 (0.3 - 4.4)	0.92
China	2/10 (20)	1.1 (0.3 - 4.7)	0.85
Guinea	17/174 (10)	0.4 (0.2 - 0.7)	<0.001
India	36/140 (26)	1.2 (0.8 - 1.8)	0.3
Kenya	26/145 (18)	0.9 (0.6 - 1.4)	0.63
Malaysia	13/39 (33)	2.1 (1.2 - 3.8)	0.01
Senegal	26/123 (21)	0.9 (0.6 - 1.4)	0.71
Thailand	9/67 (13)	0.7 (0.4 - 1.5)	0.4
Tanzania	15/87 (17)	0.8 (0.5 - 1.4)	0.44
Zambia	10/26 (38)	1.7 (0.9 - 3.3)	0.09
Zimbabwe	11/56 (20)	0.8 (0.4 - 1.6)	0.57

b.¶

Variable	N unfavorable/ N assessable (%)	Hazard Ratio (95% CI)	P value
Adherence 100%	93/921 (10)	Reference	Reference
Adherence >90% and <100%	41/234 (18)	2.3 (1.6 - 3.4)	<0.001
Adherence ≤ 90%	16/43 (37)	5.5 (3.1 - 9.7)	<0.001
HIV negative	109/1010 (11)	Reference	Reference
HIV positive	41/188 (22)	2.8 (1.8 - 4.1)	<0.001
Month 2 culture negative	100/929 (11)	Reference	Reference
Month 2 culture positive	50/269 (19)	1.8 (1.3 - 2.7)	0.001
BMI (per 5 kg/m^2 decrease)	≥ 17 kg/m^2: 109/908 (12)<br>< 17 kg/m^2: 41/290 (14)	1.5 (1.0 - 2.0)	0.03
Female	33/350 (9)	Reference	Reference
Male	117/848 (14)	1.5 (1.0 - 2.3)	0.05
South Africa	63/460 (14)	Reference	Reference
Benin	13/90 (14)	1.3 (0.7 - 2.4)	0.48
Botswana	0/7 (0)	NA	NA
China	0/7 (0)	NA	NA
Guinea	21/164 (13)	0.9 (0.5 - 1.5)	0.68
India	9/70 (13)	1.5 (0.7 - 3.1)	0.3
Kenya	14/110 (13)	1.4 (0.8 - 2.6)	0.29
Malaysia	3/17 (18)	2.5 (0.8 - 8.2)	0.12
Senegal	12/128 (9)	1.0 (0.5 - 1.9)	0.9
Thailand	2/31 (6)	0.8 (0.2 - 3.5)	0.82
Tanzania	7/55 (13)	1.2 (0.5 - 2.6)	0.69
Zambia	1/18 (6)	0.5 (0.1 - 3.9)	0.54
Zimbabwe	5/41 (12)	0.5 (0.2 - 1.4)	0.19

Supplemental Table 12. Univariate Cox proportional hazard analysis for merged MGIT and LJ culture data (as used in primary analysis)*, MGIT data only, and LJ data only in the MITT analysis population. Univariate results for **a.** experimental group study participants and **b.** control group study participants. Unadjusted analysis.

a.

Variable	N unfavorable/ N assessable (%)	Hazard Ratio (95% CI)	P value
Month 2 culture negative*	248/1478 (17)	Reference	Reference
Month 2 culture positive*	115/336 (34)	2.3 (1.9 - 2.9)	<0.001
Month 2 culture negative- MGIT data only	77/583 (13)	Reference	Reference
Month 2 culture positive-MGIT data only	141/508 (28)	2.3 (1.7 - 3.0)	<0.001
Month 2 culture negative- LJ data only	233/1386 (17)	Reference	Reference
Month 2 culture positive-LJ data only	106/319 (33)	2.2 (1.8 - 2.8)	<0.001

b.

Variable	N unfavorable/ N assessable (%)	Hazard Ratio (95% CI)	P value
Month 2 culture negative*	113/998 (11)	Reference	Reference
Month 2 culture positive*	49/285 (17)	1.6 (1.1 - 2.2)	0.007
Month 2 culture negative- MGIT data only	24/310 (8)	Reference	Reference
Month 2 culture positive-MGIT data only	42/296 (14)	1.9 (1.1 - 3.1)	0.02
Month 2 culture negative- LJ data only	108/915 (12)	Reference	Reference
Month 2 culture positive-LJ data only	43/261 (16)	1.5 (1.0 - 2.1)	0.04

* Culture positivity on either media was used for analyses with preference for solid culture if available.
