# Supplementary material for: A patient-level pooled analysis of treatment-shortening regimens for drug-susceptible pulmonary tuberculosis
Source: Nat Med. 2018 Nov 5;24(11):1708–15. doi: 10.1038/s41591-018-0224-2 (PMC6685538; doi:10.1038/s41591-018-0224-2)
Supplement: Supplementary file 2 — Reporting Summary [file 41591_2018_224_MOESM2_ESM.pdf]

nature research

Corresponding author(s): Rada Savic

Reporting Summary

Nature Research wishes to improve the reproducibility of the work that we publish. This form provides structure for consistency and transparency in reporting. For further information on Nature Research policies, see Authors & Referees and the Editorial Policy Checklist.

Statistical parameters

When statistical analyses are reported, confirm that the following items are present in the relevant location (e.g. figure legend, table legend, main text, or Methods section).

n/a	Confirmed	
☐	☒	The exact sample size (*n*) for each experimental group/condition, given as a discrete number and unit of measurement
☒	☐	An indication of whether measurements were taken from distinct samples or whether the same sample was measured repeatedly
☐	☒	The statistical test(s) used AND whether they are one- or two-sided<br>*Only common tests should be described solely by name; describe more complex techniques in the Methods section.*
☐	☒	A description of all covariates tested
☐	☒	A description of any assumptions or corrections, such as tests of normality and adjustment for multiple comparisons
☐	☒	A full description of the statistics including central tendency (e.g. means) or other basic estimates (e.g. regression coefficient) AND variation (e.g. standard deviation) or associated estimates of uncertainty (e.g. confidence intervals)
☐	☒	For null hypothesis testing, the test statistic (e.g. *F*, *t*, *r*) with confidence intervals, effect sizes, degrees of freedom and *P* value noted<br>*Give P values as exact values whenever suitable.*
☒	☐	For Bayesian analysis, information on the choice of priors and Markov chain Monte Carlo settings
☐	☒	For hierarchical and complex designs, identification of the appropriate level for tests and full reporting of outcomes
☐	☒	Estimates of effect sizes (e.g. Cohen's *d*, Pearson's *r*), indicating how they were calculated
☐	☒	Clearly defined error bars<br>*State explicitly what error bars represent (e.g. SD, SE, CI)*

Our web collection on statistics for biologists may be useful.

Software and code

Policy information about availability of computer code

Data collection	Data collection and formatting was performed using the R Statistical Software version 3.4.3.
Data analysis	All data analysis was performed using the R statistical Software version 3.4.3.

For manuscripts utilizing custom algorithms or software that are central to the research but not yet described in published literature, software must be made available to editors/reviewers upon request. We strongly encourage code deposition in a community repository (e.g. GitHub). See the Nature Research guidelines for submitting code & software for further information.

Data

Policy information about availability of data

All manuscripts must include a data availability statement. This statement should provide the following information, where applicable:

- Accession codes, unique identifiers, or web links for publicly available datasets
- A list of figures that have associated raw data
- A description of any restrictions on data availability

The standardized data for the OFLOTUB (ClinicalTrials.gov number, NCT00216385) , REMoxTB (NCT00864383), and RIFAQUIN (ISRCTN number, 44153044) trials that support the findings of this study are publicly available to qualified researchers through the Platform for Aggregation of Clinical TB Studies (TB-PACTS, https://c-path.org/programs/tb-pacts/). The DMID 01-009 (NCT00130247) data that support the findings of this study are available from the Tuberculosis Research Unit at

Cape Western Reserve University but restrictions apply to the availability of these data, which were used under agreement for the current study. Data are however available from the authors upon reasonable request and with permission from the Tuberculosis Research Unit.

Field-specific reporting

Please select the best fit for your research. If you are not sure, read the appropriate sections before making your selection.

☒ Life sciences ☐ Behavioural & social sciences ☐ Ecological, evolutionary & environmental sciences

For a reference copy of the document with all sections, see nature.com/authors/policies/ReportingSummary-flat.pdf

Life sciences study design

All studies must disclose on these points even when the disclosure is negative.

Sample size	No sample size calculation was performed. This was a meta-analysis that used all available data from four previously published clinical trials.
Data exclusions	The analysis focused on 6-month standard of care regimens and 4-month experimental regimens to identify populations eligible for short course tuberculosis treatments. Therefore, data from the 6-month experimental arm in the RIFAQUIN trial was not included in the analysis.
Replication	All data management and harmonization notes are available in the Supplementary Information. Analysis code for the meta-analysis that was performed in the R statistical software is clearly annotated for reproducibility.
Randomization	Randomization and blinding was performed in the parent trials that were previously published.
Blinding	Randomization and blinding was performed in the parent trials that were previously published.

Reporting for specific materials, systems and methods

Materials & experimental systems

n/a	Involved in the study
☒	☐ Unique biological materials
☒	☐ Antibodies
☒	☐ Eukaryotic cell lines
☒	☐ Palaeontology
☒	☐ Animals and other organisms
☐	☒ Human research participants

Methods

n/a	Involved in the study
☒	☐ ChIP-seq
☒	☐ Flow cytometry
☒	☐ MRI-based neuroimaging

Human research participants

Policy information about studies involving human research participants

Population characteristics	Population characteristics are available in Table 1 of the manuscript. In summary, participants were recruited in 16 countries with most in Sub-Saharan Africa. Majority of the participants were Black males with a median age of 30 years. Only 12% of the population were co-infected with HIV.
Recruitment	Recruitment was performed in the parent trials that were previously published.
